# Support Needs and Available Resources for School‐Aged Siblings of Children With Disabilities: A Mixed Methods Study

**DOI:** 10.1111/jar.70190

**Published:** 2026-01-30

**Authors:** Linda K. M. Veerman, Agnes M. Willemen, Suzanne D. M. Derks, Anjet A. J. Brouwer‐van Dijken, Paula S. Sterkenburg

**Affiliations:** ^1^ Department of Clinical Child and Family Studies; LEARN!; Amsterdam Public Health Vrije Universiteit Amsterdam Amsterdam the Netherlands; ^2^ Brussenboek.nl Bilthoven the Netherlands; ^3^ Sibling Carers Community BRUS Utrecht the Netherlands; ^4^ Bartiméus Doorn the Netherlands

**Keywords:** children, intellectual disability, interviews, review, siblings, support needs, support resources, visual impairment

## Abstract

**Background:**

This study aimed to provide insight into the support needs of siblings (6–12 years) of children with intellectual disabilities and/or visual impairments, and in the match with sibling support resources that are available in the Netherlands and Belgium.

**Method:**

Semi‐structured interviews about support needs were conducted with 13 siblings. A grey document review identified 255 organisations and 134 books, texts and media sources that offer (indirect) support to siblings. Thematic analysis was conducted on the interview data and found resources.

**Results:**

Siblings described diverse challenges and support needs, such as social and emotional support, receiving quality time and attention, and contact with peers. The found resources were fragmented, mainly focused on a broader target group, and only partially matched siblings' needs. Some support needs, such as learning to deal with their siblings' behaviour, were insufficiently addressed.

**Conclusion:**

These insights can help better align sibling support needs with available resources.

## Lay Summary

- Siblings are part of the social context of the family. Therefore, supporting siblings can benefit the whole family, including the children with a disability. In this research we studied the support needs and support resources for siblings of children with intellectual disabilities and/or visual impairments.
- Interviews showed that siblings need support with the challenges and emotions they experience in their family (e.g., dealing with the behaviour of their sibling or talking to someone about their worries).
- A website search in the Netherlands and Belgium showed that several support resources for siblings are offered. However, these resources are scattered across the care system and not easy to find. Also, some support that siblings wish to receive is barely offered.
- This study outlines the support siblings seek and offers examples of how it can be provided. This can help organisations offer and families find the support siblings need.

## 1 Introduction

### 1.1 Background

In a recent study, adult siblings of persons with a chronic condition reported that, as a child, they felt ‘not seen’ and missed support with the psycho‐emotional difficulties that they experienced (Hanvey et al. 2022). Yet, the experiences of siblings of children with disabilities include a range of conflicting emotions (e.g., Moyson and Roeyers 2012). For example, most siblings of children with disabilities have a strong bond and experience great loyalty (Vella Gera et al. 2021), but also face jealousy and sadness (Haukeland et al. 2015). Siblings of children with complex care needs or neurodevelopmental disorders are confronted with situations that their peers cannot relate to, such as worries about their sibling, which may lead to feelings of loneliness and participation difficulties (Pavlopoulou et al. 2025; Woodgate et al. 2016). Moreover, siblings of children with chronic conditions have an increased risk of mental health problems (Martinez et al. 2022) and siblings of children with disabilities have poorer school and social functioning (Goudie et al. 2013).

The experiences and well‐being of siblings of children with neurodevelopmental disorders are associated with a range of risk and resilience factors on the individual, family and context level (Kovshoff et al. 2017; Wolff et al. 2022, 2025). One of these factors is social support, which includes informal support from family members or friends and formal support from professional caregivers or teachers. Support can be defined as ‘resources and strategies that aim to promote the development, education, interests, and personal well‐being of a person and that enhance individual functioning’ (Luckasson et al. 2002, 151). It is positively related to resilience factors in siblings of children with neurodevelopmental disorders, such as coping skills and self‐concept, and negatively related to psychosocial problems, loneliness, and stress (Kirchhofer et al. 2022). Thus, support can enhance siblings' well‐being and prevent negative mental health outcomes.

To effectively support siblings of children with disabilities, it is important to understand their specific support needs. Several recent studies investigated these needs. First, a recent review identified support needs of siblings of persons with developmental disabilities within studies about siblings' perceptions of their quality of life (Múries‐Cantán et al. 2023). These needs include emotional support, being involved, receiving information, private time, and attention from their parents. Second, Joosten et al. (2019) conducted a survey and interviews about support needs among siblings (12–18 years) of children with chronic conditions. This study showed that teenage siblings are interested in meeting peers and receiving online support on various topics, such as their concerns related to their sibling. Third, retrospective interviews with adult siblings of persons with disabilities or chronic illnesses revealed unmet support needs of siblings during childhood (Hanvey et al. 2022). This includes validation of their experiences and needs, and the opportunity to exchange experiences with peers. Finally, Meltzer (2021) conducted interviews with providers of support for siblings of persons with disabilities about their perspective on sibling support aims. They mentioned siblings' need for recognition and validation, social–emotional support, and knowledge and skills. Although the methods of these studies are varied, the shared conclusion is that siblings need support and that these needs are diverse.

Several interventions that contribute to meeting the needs of siblings of children with chronic conditions have been developed (McKenzie Smith et al. 2018; Wolff et al. 2023). For example, a group parent‐sibling intervention for families with a child with a chronic condition showed to reduce siblings' mental health problems and improve parent–child communication, but not compared to a waitlist control group (Kirchhofer et al. 2025). A 10‐week peer support group for siblings of children with autism showed to have significant, positive effects on siblings' externalising behaviour and coping skills in mental health in comparison to an attention‐only control group (Jones et al. 2020). A realist review conducted by Marquis et al. (2022) reported that siblings of children with intellectual or developmental disabilities seem to benefit from validation of their needs and role, quality time with their parents, and respite, such as relaxing activities away from the care situation. Siblings of children with intellectual disabilities that participated in interventions particularly valued meeting peers and receiving support in a playful manner (Carter et al. 2016).

However, the interventions that are researched are not all available in daily practice. Support for siblings of persons with disabilities appears to be underrecognized and lacks financial support by governments (Meltzer 2022), even though the Convention on the Rights of Persons with Disabilities (United Nations 2006, art. 23) requires nations to provide early support to the families of children with disabilities, including siblings. Also, studies conducted in hospitals and healthcare organisations in Canada (Nguyen et al. 2021) and the United States (Mooney‐Doyle et al. 2022), and in local authority services in the United Kingdom (Taylor et al. 2025) revealed that limited support resources directly targeted the needs of siblings of children with serious illnesses or intellectual disabilities.

### 1.2 The Current Study

Although several studies have researched support needs and available support programs, some topics have not yet been fully covered. First, previous studies have focused on siblings' experiences of growing up with a sibling with a disability (e.g., Moyson and Roeyers 2012), but to our knowledge, no scientific studies have specifically included young (< 12 years) siblings' perspectives on their own support needs and preferences regarding how and from whom they want to receive support. The first aim of this study was therefore to gain a better understanding of young siblings' needs. Interviews were conducted with school‐aged siblings of children with intellectual disabilities and/or visual impairments (hereafter referred to as ‘siblings’) about the challenges they face, the kinds of support they wish to receive, and the persons they want to receive support from. It was decided to include both siblings of children with intellectual disabilities and visual impairments. There is a large comorbidity between intellectual disability and visual impairment (Van Splunder et al. 2005). At the same time, children with visual impairments often have additional developmental disabilities, which may not always be diagnosed in early childhood (Chokron et al. 2020; De Verdier et al. 2018). Moreover, siblings of children with visual impairments are underrepresented in sibling research (Veldhorst et al. 2023), while the difficulties they face are evident, such as experiencing feelings of sadness, anxiety or guilt towards their sibling or the disability (Şengül Erdem et al. 2024).

Second, little is known about the sibling support that is available in the Netherlands and Belgium, and across different support settings (e.g., disability care, social services). The second aim of this study was to provide an overview of direct (targeted at siblings themselves) and indirect (targeted via others, such as parents) support resources for siblings of children with disabilities. A systematic grey document review was conducted to detect resources specifically for siblings of children with intellectual disabilities and/or visual impairments, or for a broader target group that contains these siblings. Support targeted at young carers (i.e., children that grow up with a family member with care needs) was thus also included. An overview was created of the types of organisations, types of resources, target groups, and their accessibility. This also provides valuable insights for future international studies into available sibling support resources.

Finally, this study aimed to explore the match between the available support resources and the support needs that siblings have. In this matter, the representation of the support needs that siblings mentioned in the interviews within the available resources was studied. Altogether, this study provides families, care professionals, policy makers, and researchers with an overview of the available support resources and new insights that could guide the support they offer to siblings.

## 2 Methods

The current study used a mixed methods design combining individual semi‐structured interviews (Part 1) with a grey document review of sibling support resources (Part 2). The study protocol has been pre‐registered on OSF.io (Veerman et al. 2022). The methods of the two parts are described subsequently.

### 2.1 Part 1: Support Needs Interviews

#### 2.1.1 Participants

The sample comprised 13 children aged 6 to 12 years (*M* = 9.23, SD = 2.13) from Belgium (*n* = 2) and the Netherlands (*n* = 11). Most participants never received formal support before (*n* = 8). Some families had a non‐European migration background (*n* = 3). Two participants had a neurodevelopmental disorder themselves (attention deficit disorder; developmental language disorder). One participant had a brother who lived in a residential care facility; the other's siblings lived at home. Table 1 provides an overview of the obtained demographics.

**TABLE 1:** Sample demographics (*N* = 13).

No.	Gender	Age	Birth order	Sibling^a^	Diagnose sibling	Family composition
1.^b^	Girl	12	Oldest	Brother	Severe intellectual disability; autism	Mother, father, two children
2.	Boy	9	Middle	Sister	Rare genetic disorder; severe intellectual disability	Moter, father, three children
3.^c^	Girl	6	Twins	Brother	Blindness	Mother, father, two children
4.	Boy	8	Oldest	Brother	Moderate/severe intellectual disability; autism	Mother, father, two children
5.	Boy	12	Oldest	Brother	Rare genetic disorder; moderate/severe intellectual disability	Mother, father, two children
6.	Girl	11	Oldest	Brother	Profound intellectual and multiple disabilities	Mother, father, two children
7.	Girl	10	Youngest	Sister	Mild intellectual disability; cerebral visual impairment; motor impairment	Mother, father, two children
8.	Boy	11	Youngest	Sister	Rare genetic disorder; moderate/severe intellectual disability; autism	Mother, father, three children
9.	Girl	6	Youngest	Brother	Rare genetic disorder, cerebral visual impairment; developmental delay	Mother, stepfather, two children
10.	Girl	8	Oldest	Sister	Rare genetic disorder; profound intellectual and multiple disabilities	Mother, father, three children
11.^d^	Boy	7	Youngest	Brother	Rare genetic disorder; mild/severe intellectual disability; autism; epilepsy	Mother, father, three children
12.^d^	Boy	9	Middle	Brother	Rare genetic disorder; mild/severe intellectual disability; autism; epilepsy	Mother, father, three children
13.^c^, ^e^	Boy	11	Youngest	Brothers	Progressive eye disease; autism (only oldest sibling)	Mother, father, three children

^a^ This indicates the sibling with a disability.

^b^ The brother of participant 1 lives in a residential care facility.

^c^ Participants 3 and 13 have a neurodevelopmental disorder themselves (attention deficit disorder; developmental language disorder).

^d^ Participants 11 and 12 are brothers.

^e^ Participant 13 has two brothers with a disability.

#### 2.1.2 Procedures

Ethical approval was obtained from the Ethics Committee of the Faculty of Behavioural and Movement Sciences of the Vrije Universiteit Amsterdam (VCWE‐2022‐173). Both parents of the participants and the children aged 12 years signed informed consent forms. Explicit consent was obtained for publishing any quotes from the interviews. All children provided assent to being interviewed.

Siblings were asked to participate in a study about support needs through flyers on social media, parent gatherings, and in newsletters. In total, 22 families showed interest in the study, of which 21 were eligible for participation. The researcher called these parents to provide further information and acquire demographic information. Then, from these families, a diverse sample was selected based on the provided information on age, gender, disability, country, and cultural background. First, an initial sample of eight participants was selected. Then, based on thematic summaries of the interviews, it was determined that saturation was not yet reached. Therefore, new participants were selected one by one until saturation was reached. One final participant was included to confirm that no new themes arose.

In 2023, a researcher (LV; *n* = 9) or research assistant (*n* = 4) conducted one‐on‐one semi‐structured interviews at the participants' homes (*M* = 43 min). The research assistant was a sibling herself and mentioned that in the interview to build rapport. The child chose the room in which the interview took place, and the parent was present at home, but not in the same room. The interview consisted of three parts, as described in Table 2. The researchers used an interview protocol including pre‐defined questions, guidance for non‐defined follow‐up questions, visual aids (i.e., forms and communication boards), and instructions based on guidelines for interviewing children (Irwin and Johnson 2005; O'Reilly et al. 2013). The protocol was developed by the authors, including a sibling research partner (ABvD), and in consultation with an expert (sounding board) group of parents, siblings and researchers in the field. The visual aids were based on previous sibling research (e.g., Moyson and Roeyers 2012). Prior to data collection, the interviewers tested and reviewed the protocol with three siblings and another child in the age range. These interviews were not included in this study.

**TABLE 2:** Interview procedures and visual aids.

Part	Steps	Description	Visual aid^a^
1.	a.	Write down names of persons that are important in your life.	Visual aid A: Form with 14 categories of support providers (e.g., family members, friends, teachers).
	b.	Share something about your family. Tell about what you like about your sibling or enjoy doing together.	None
2.^b^	a.	Describe a difficulty you face with your sibling with a disability. This can be related to the examples shown on the pictograms or another example.	Visual aid B: Communication board with pictograms of 12 categories of difficulties (e.g., worrying, being hurt by my siblings, feeling invisible)
	b.	Share how you are or could be helped with the described difficulty. Name your own ideas or elaborate on an example from the pictograms.	Visual aid C: Communication board with pictogram examples within four categories: (1) *Getting information*, (2) *Talking about it*, (3) *Learning how to deal with it*, (4) *Something nice especially for me*.
	c.	Share from whom you receive or would wish to receive this support.	Visual aid A is used again
3.	a.	Share any other examples of support on Visual aid C that you would wish to receive, regardless of experiencing difficulties.	Visual aid C is used again
	b.	Anything else you want to share.	None

^a^ Translated versions of the visual aids are presented in Supporting Information S1.

^b^ The three steps in part two were repeated until the participant could not name new examples.

At the end of each interview, a brief evaluation took place, and participants received a small gift. All interviews were audiotaped using a voice recorder. Remarks or potential interviewer biases were reported in a logbook (e.g., whether the sibling felt at ease) and read by the coders to provide context during the qualitative coding of the data. Individual member checks were performed in a video call one week after each interview, discussing a summary with pictograms. This only provided minor changes in some cases (e.g., removal of one of the support preferences). Also, participants could provide written responses to a summary of overall results. Three participants responded that results were recognisable; they had no additional comments.

#### 2.1.3 Data Analysis

The data was analysed using semantic, inductive, codebook thematic analysis (Braun and Clarke 2022) in Atlas.ti 22 (ATLAS.ti Scientific Software Development GmbH 2021). First, the interviews were transcribed by research assistants. Next, five transcripts were coded independently by two researchers (LV and a research assistant that was not involved in conducting the interviews). Based on all relevant information from the interviews, the first version of the codebook was created in consultation with an experienced qualitative researcher (PS). Using this codebook, all transcripts were then coded by one or two (> 60%) researchers. In this process, the two researchers further developed the codebook, adding new codes and clustering the codes in themes. Then, using a back‐and‐forth approach, the codebook was adapted until it covered all the relevant data. Throughout this process, the codebook was reviewed by the research assistant involved in conducting the interviews (adult sibling), experienced qualitative researchers (PS, AW) and a sibling research partner (ABvD). Finally, to increase the reliability of the codebook, all transcripts were coded by a third independent research assistant (master's student Educational Sciences, with experience in youth care). Accordingly, it was concluded that the themes were clear and covered the data, but that the codes within these themes could not all be distinguished and should not be reported as subthemes but rather as examples within the description of the themes. For the same reason, a few themes were merged. Finally, the authors selected illustrating quotes from the interviews that were translated by the first author (LV) from Dutch to English. A bilingual independent researcher, who is a native English speaker, checked the translated quotes for accuracy.

### 2.2 Part 2: Support Resources Review

The PRISMA guidelines (Page et al. 2021) have been followed, as far as possible with the type of documents that were reviewed.

#### 2.2.1 Eligibility Criteria

Resources were eligible for inclusion when they met the following criteria: (1) available in Dutch, (2) targeted at siblings or young carers in general, directly or indirectly through their parents or professionals (e.g., exclusion when it is about siblings of children without a disability), (3) targeted at (persons involved with) children aged 6–12 years (e.g., exclusion when about adults or adolescents), (4) targeted at (persons involved with) siblings of children with intellectual disability and/or visual impairment specifically or a broader range of diagnoses that include intellectual disability or visual impairment (e.g., exclusion when it is specifically for siblings of children with cancer), (5) can be perceived as (directly or indirectly) supportive in a broad sense, according to the definition in the Introduction (e.g., exclusion when it only includes minimal information, such as on prevalence). Due to cultural, societal, and organisational differences, organisations in countries in South America in which Dutch is an official language (e.g., Suriname and Curaçao) were not included.

#### 2.2.2 Search Strategies

A range of search strategies were used, based on grey literature search examples (Adams et al. 2016; Godin et al. 2015) and in consultation with a librarian of Vrije Universiteit Amsterdam. First, grey literature databases and Dutch webpages of organisations that produce grey literature were searched (see Supporting Information S2). For each webpage a tailored search procedure was used (Stansfield et al. 2016). Second, websites of care organisations in the Netherlands and Belgium were searched, using either the website's search bar or Google to search on the site. For these two strategies we used the separate search terms ‘brother’, ‘sister’ and ‘sibling’. Third, a Google search was conducted with the search string ‘brother | sister | sibling’ + ‘~intellectual | ~visual + ~disability’. By using this search string, results were also included for synonyms of ‘intellectual’, ‘visual’ and ‘disability’.

This part of the search was performed in two phases from December 2022 until December 2023. First a list of relevant organisation websites was computed by two researchers (LV and a research assistant), based on national websites that provide an overview of care organisations or (young) carer organisations. This included care organisations for residential care, rehabilitation, and clinical treatment, but not daycare only. Next, four researchers (LV and three research assistants) conducted the search and registered all found sources in an Excel file. For organisation websites with more than 20 hits, only the relevant sources were registered. For database searches and the Google search, the first 10 pages (100 results) and all relevant results until no more relevant results were shown were registered. Doubles (identical URLs) and sources that were clearly irrelevant based on the title were marked and deleted in the next step of the procedure.

Next, three additional search strategies were used. A call was posted on Instagram, Facebook, LinkedIn and in the newsletter of the overarching research project to fill out known support resources in an anonymous online form (Qualtrics 2022). Input was gathered from January until April 2024. Only resources that were new and relevant were added to the list and screened for eligibility. Fifth, snowballing was used to include resources that were referenced in the found sources. Finally, missed resources that were known by an expert by experience (ABvD) or the researcher (LV) were added. Unlike what was planned in the preregistration, other experts in the field were not asked to add missed resources because of time constraints and already having received responses from experts to the social media call.

#### 2.2.3 Data Selection, Extraction and Analysis

From December 2023 until April 2024, three researchers (LV and two research assistants) screened the sources for eligibility. In this data selection procedure, the researchers screened the full texts based on the listed criteria. For books, series and films, only the descriptive information was screened. The reason for exclusion was registered.

From March until June 2025, a researcher (LV) and five research assistants extracted relevant data from the sources, using a digital extraction form (Qualtrics 2022) (see Supporting Information S3). At the same time, snowballing was performed and new, relevant organisations or resources that were referred to on the found websites were added. Webpages and sources from the same organisation were clustered, so that one data extraction form was filled out per organisation. The first part of the extraction form included information about the organisation and the types of support that the organisation offers. Next, for each type of support, additional information was filled out, clustering the resources of the same type within each organisation. In addition, deductive thematic analysis of the texts of the resources was conducted within the extraction form, using the themes about challenges and support kinds from the interviews (Part 1). Herein, it was only analysed whether the themes were mentioned in the text and not if the resource provided support in relation to the themes. Because books, news articles, research texts or reports, and other media were often not offered by specific organisations, separate, slightly adapted extraction forms were used to capture relevant information from these types of sources.

The data that was inserted in Qualtrics was exported to IBM SPSS Statistics (Version 29) to analyse frequencies on the organisation level and on the resource level. Open‐ended input was clustered in existing or new categories or were used as examples.

#### 2.2.4 Quality Assessment and Risk of Bias

To reduce the risk of bias, two researchers computed the Google search at the same time and location. In the data selection process, the three involved researchers screened 100 sources individually and then discussed the decision procedure before continuing. Also, 10% of the sources were screened for eligibility by two independent researchers. The discordant decisions were discussed until a decision was reached. For all sources, double extraction was used, where one research assistant filled out the form and the other checked and corrected the form. A test set was extracted by all research assistants; based on this, the extraction form was improved. Regular meetings were held to discuss unclarities. Due to limited staff capacity, the study was paused for 9 months. Previously selected resources were checked and those that could no longer be found online were deleted. Additional resources were included using snowballing. Unlike the pre‐registration, it was decided not to include a quality assessment of the found sources because of the number and diversity of resources.

## 3 Results

### 3.1 Part 1: Support Needs Interviews

**FIGURE 1:**
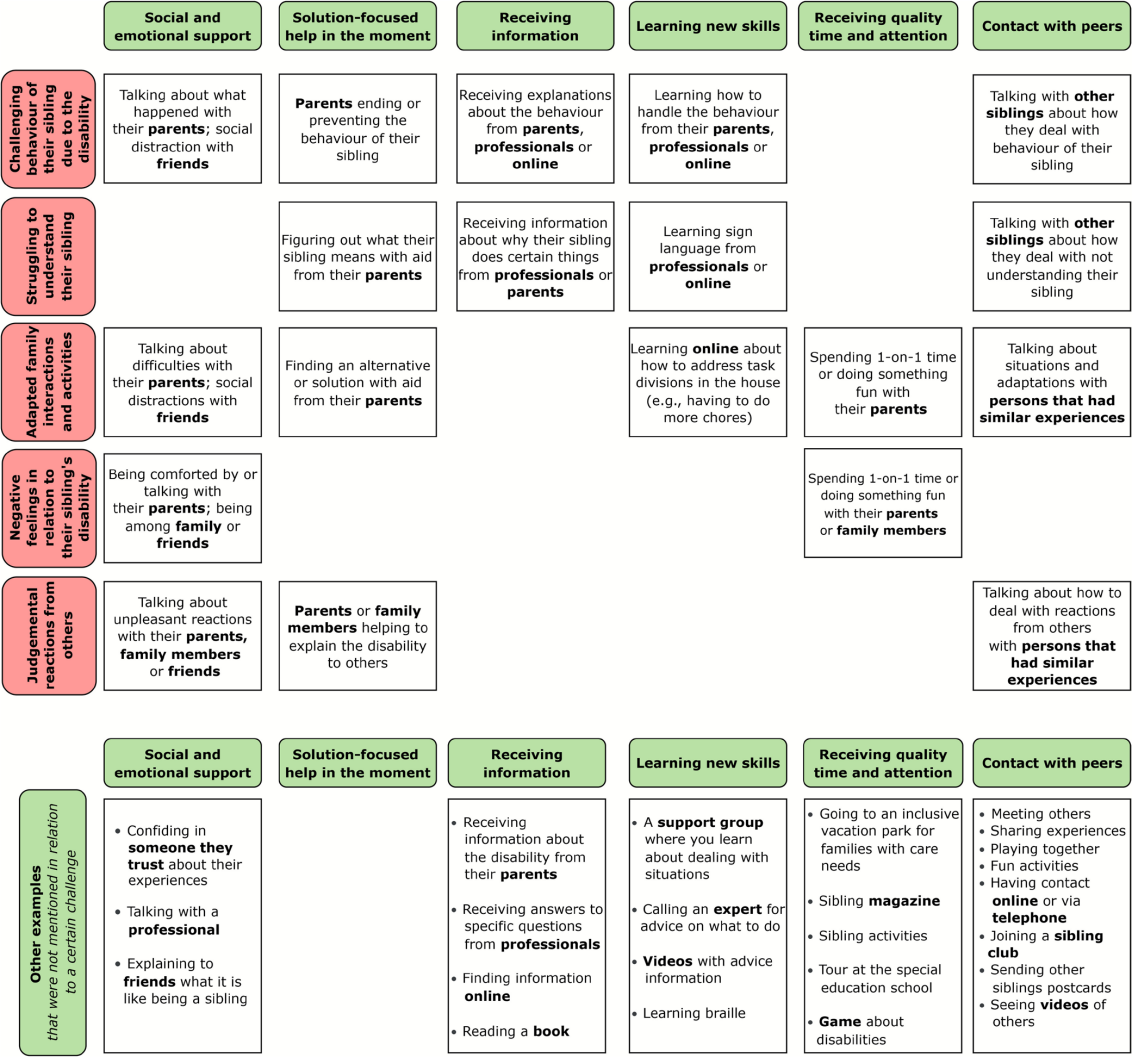
Overview of themes and examples regarding experienced challenges, kinds of support and support persons. This figure only includes examples that were given by the participants of this study. Other examples of support are possible. Blank spaces indicate that no examples related to these challenges and support kinds were given.

The results from the interviews are described by themes in three categories: (1) challenges that siblings experience, (2) kinds of supports that siblings (wish to) receive, and (3) persons that siblings (wish to) receive support from. In addition, preferences in the means of support are reported. Examples and co‐occurrences are described in Figure 1.

#### 3.1.1 Which Challenges Do Siblings Experience?

The challenges that participants mentioned about having a sibling with a disability can be summarised in five themes. The first theme comprised *struggling to understand their sibling*, which includes challenges in communicating with their sibling and difficulties in understanding the reasons for their siblings' behaviour. Second, they mentioned *challenging behaviour of their sibling due to the disability* that disrupts the participants' activities, annoys, or hurts them. Examples include yelling, odd behaviour, claiming or fighting over things, being hit or pushed, or getting their hair pulled. Third, most participants mentioned *adapted family interactions and activities*, such as activities that are not possible to do together as a family, unbalanced division of parental attention, adaptations to the house or activities, not being able to play together as siblings, and having to help more or adapt their own behaviour because of their sibling's needs. A few participants mentioned *judgemental reactions from others*, such as people staring at their sibling, expressing a lack of understanding, or even bullying them. Finally, participants mentioned *negative feelings in relation to their sibling's disability*, such as sadness, anger, irritation, shame or worry. For example, participants mentioned feeling irritated or upset by the behaviour of their sibling or worried that something might happen to their sibling.

Next to the described challenges, participants also mentioned positive aspects. These include positive experiences, thoughts and feelings, positive traits of their sibling, such as things that their sibling can do despite their disability, and warm emotions towards their sibling. It stood out that many participants mentioned that they enjoy doing those things that their sibling enjoys, such as cuddling or playing with something their sibling likes.

#### 3.1.2 What Kind of Support Do Siblings Want to Receive?

The kinds of support that were mentioned by the participants can be categorised into six themes. The first theme comprised *social and emotional support*, such as someone being available to talk about thoughts, feelings and experiences, being comforted when something happened, being accompanied by others, or having social distractions. Participants mostly mentioned emotional support as something that they already receive from their parents. A few mentioned they prefer not to talk about difficulties and feelings, but rather seek distraction, for example by playing with friends or watching videos. A 6‐year‐old girl described that she asks her mother for support when something happens between her and her brother:

Uhm, today he was angry with me, because I was trying to close his zipper and then he said “you did it wrong”. But he said that very angrily, and then I cried on the couch […] then I stood up again and I went to mommy. And then mommy was still in the shower and then I said: ‘Mommy’, I said: ‘Mommy I did it right, but [name brother], but [name brother] does, uhm, [name brother] says that I did it wrong, but that is not true.’ [Interviewer: ‘Yes, that is not so nice. So you went to mommy to talk about it. And what happened next?’] ‘And then it was all okay again.’ (Participant 9)

Second, participants mentioned that *solution‐focused help in the moment* can be helpful. This includes receiving help in a specific situation or searching for a solution or alternative with someone. For example, participants said that it helps when their parents step in to stop their sibling's behaviour. However, siblings also mention coping strategies that they use themselves, such as finding a solution themselves or getting out of the situation. The third theme comprises *receiving information*. This includes information about the disability and its causes, and explanations for their sibling's behaviour. Some participants felt they already knew a lot and did not need more information. Fourth, participants mentioned *learning new skills*, such as how to handle their sibling's behaviour or specific situations, or disability‐related skills, such as sign language or braille. For example, an 8‐year‐old boy said:

Well, I really want to learn, because look, when he [his brother] for example does something else, not hits me, but kind of pulls on me… then I don't understand what I need to do. So, then I really want to learn how I uhm, need to uhm, can deal with that. (Participant 4)

The fifth theme comprises *receiving quality time and attention*, which includes mostly one‐on‐one attention from their parents, such as doing something fun with their parent(s) or receiving exclusive attention. Specifically, some participants mentioned that quality time without their sibling is needed. A few would like sibling‐specific resources, such as postcards, magazines for siblings, or special events.

Finally, most participants said they would like *contact with peers* (e.g., children who also have a sibling with a disability) to exchange experiences or have fun together, such as playing or going on trips. Some mentioned wanting to join a sibling club. Others felt no need for peer contact, unless it could help others. An example of how peer contact could be helpful was given by a 10‐year‐old girl:

Talk with other siblings about how they deal with it. […] Then I tell how I do it, and you tell how you do it. And then we exchange that and then maybe we can help each other. (Participant 7)

Almost all participants said they did not want or need support with some of the challenges they faced. Some coped on their own by seeking distraction, getting out of the situation, thinking positively, or understanding the situation. Others mentioned they had gotten used to certain difficulties, such as their sibling's behaviour. However, most siblings did say they wished to receive some of the suggested support resources, despite indicating that they do not need support with the challenges they faced. Some siblings said that they do not know what could help or think nothing could help with the challenges they experience. A 7‐year‐old boy for example said:

Actually, I can't do anything about it. I just do other things. Or just things with other people. […] Actually, well, no one can really help me with that exactly. Because they cannot make his disability go away. (Participant 11)

#### 3.1.3 How and From Whom Do Siblings Want to Receive Support?

Participants shared preferences of how and from whom they would like to receive support. Some preferred digital options (e.g., (video)calls, videos, games, or social media), whereas others favoured in‐person support. Few chose books or magazines. Participants also emphasised the importance of feeling understood, age‐matched peer groups, and support tailored to their specific situation and their siblings' disability. Finally, some siblings mentioned that support should be combined with fun, so that it does not feel ‘too serious’. This is illustrated by an 11‐year‐old girl:

Well, because uhm, I'd like it if, that I am not, you know [talking] all the time… that there would also be something fun, because it is also hard to talk about it sometimes, and then I also have a bit of distraction. (Participant 6)

Parents were mentioned most often as providers of all kinds of support. Sometimes participants mentioned both parents, other times they mentioned their mother or father specifically, mothers being mentioned more often than fathers (e.g., ‘*Most often with mommy, because* [name stepfather] *often needs to work*’). A few also mentioned friends (from school or the neighbourhood), mainly for emotional support and distraction. Some mentioned grandparents, their other siblings, or other relatives (e.g., aunts, cousins) for social and emotional support, solution‐focused help and quality time and attention. About half named professionals, such as caregivers of their sibling or experts on the subject, mostly for information or skills training, and sometimes as someone to talk to. Teachers were not mentioned as a source of support with challenges in relation to their sibling.

### 3.2 Part 2: Support Resources Review

**FIGURE 2:**
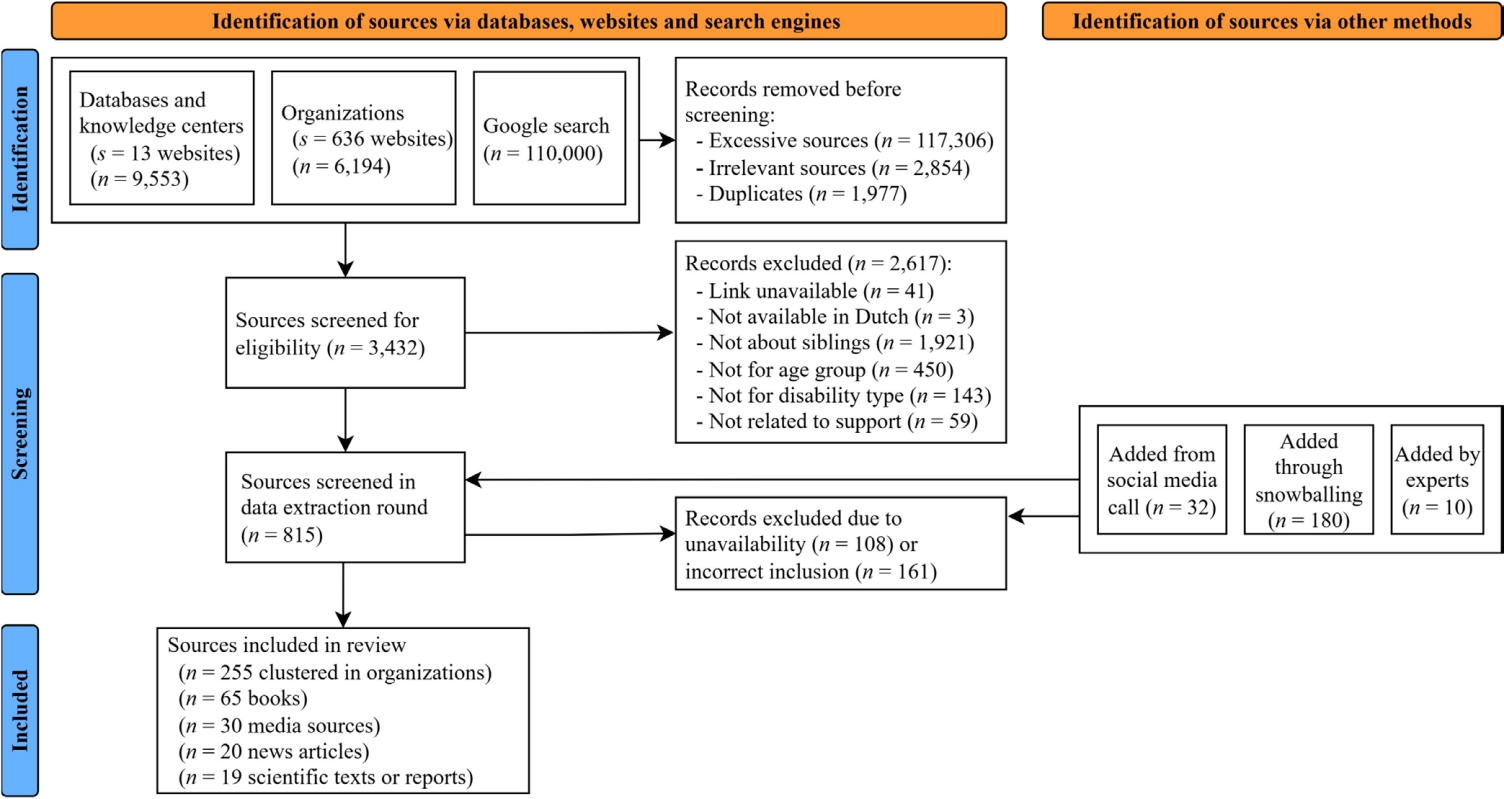
PRISMA flow diagram of the selection process.

The PRISMA flow diagram (Page et al. 2021) in Figure 2 provides an overview of the selection process and included resources. Of all the preselected websites of relevant care organisations (*N* = 525), only 10% included a support resource for siblings. For the young carer organisations (*N* = 111), this was 69%. An overview of the included organisations and resources is provided in Supporting Information S4.

#### 3.2.1 Characteristics of Included Resources

##### 3.2.1.1 Types of Organisations

**FIGURE 3:**
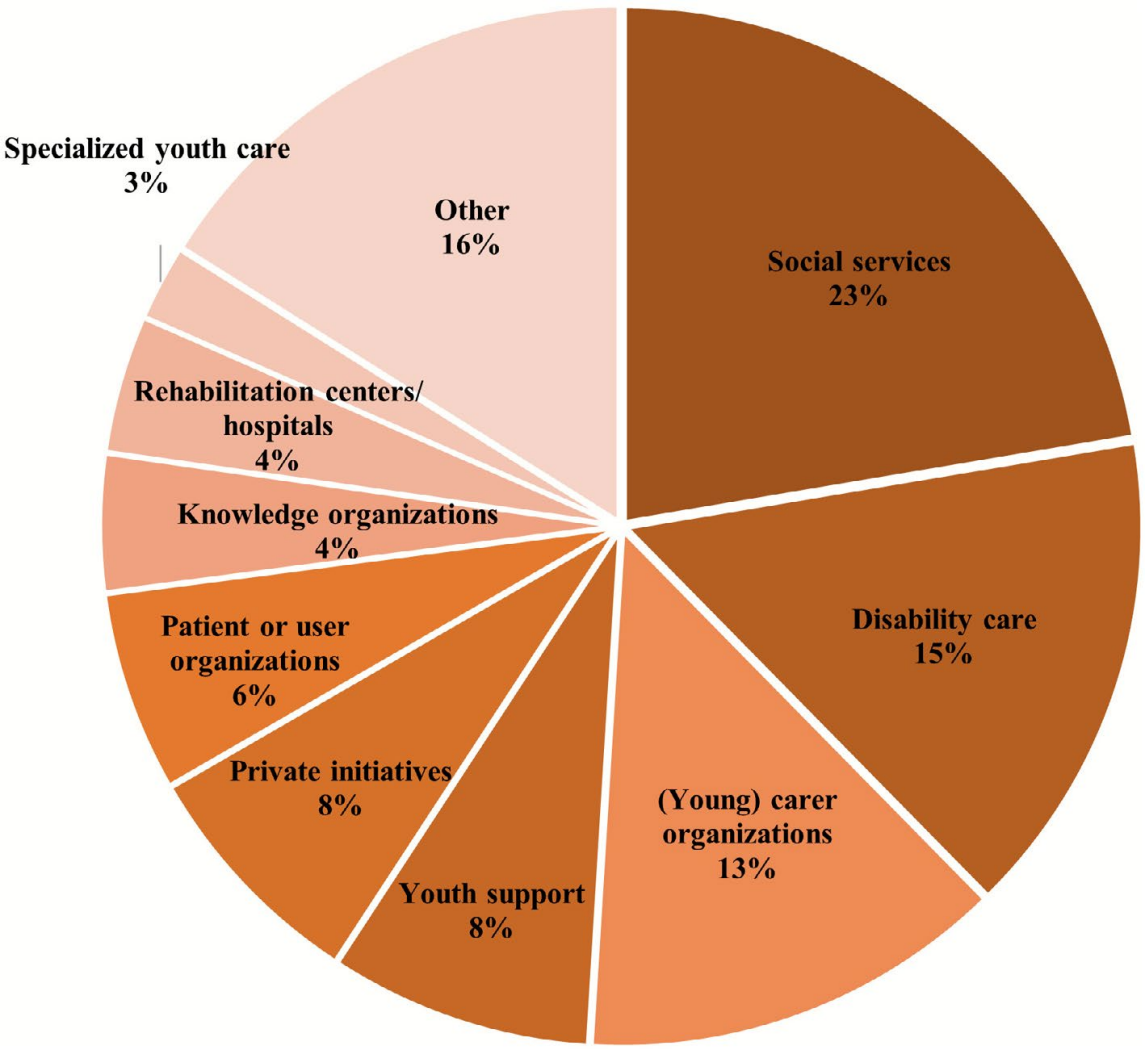
Overview of the types of organisations that offer support to siblings. Other organisations include, for example, coaches, charities, research or study projects, and volunteer organisations.

Of the 255 organisations that provided support resources for siblings, 220 were from the Netherlands (86%) and 35 from Belgium (14%). The organisations represented different sectors in the support system (see Figure 3). Most were categorised as social services or specific forms of social services, such as (young) carer organisations or youth support. These organisations are usually funded by municipalities. Some support was offered by disability care organisations. Other resources were scattered over different types of websites or organisations, such as private initiatives or charities.

##### 3.2.1.2 Types of Resources

Table 3 provides an overview of the types of resources that were found within the included organisations. The found resources were categorised in pre‐defined types, which were divided into four overarching categories: information sources, fun activities, interventions or support groups/persons, and other types (e.g., gifts, videos). Around a third of the organisations only included one type of resource (*n* = 84), whereas others provided two (*n* = 58), three (*n* = 38), four (*n* = 29), five (*n* = 26), or six to nine (*n* = 20) types of resources for siblings. The resources that were offered most often were references to other resources, information about siblings or young carers, and fun activities.

**TABLE 3:** Information about the resources offered by organisations (*N* = 255).

Type of resource	Number of organisations	Target groups (*n*)^b^	Examples
**Information sources**			
References to other resources	99 (39%)	Young carers (52); siblings (47); parents (21); care professionals (11); broad audience (10); teachers (2); not specified (1)	– References to books for siblings – References to videos or tv series – References to other organisations that offer support
Information about siblings or young carers	96 (38%)	Young carers (44); care professionals (23); siblings (20); parents (17); broad audience (12); teachers (2); not specified (2); personal network of young carers (1)	– Information folder about the impact of being a young carer or sibling – Explanation about the term ‘young carer’ or ‘brus’^c^ – Information about emotions siblings can experience
Tips for or about siblings or young carers	63 (25%)	Young carers (36); parents (17); care professionals (14); siblings (9); broad audience (2); teachers (1); friends (1); municipalities (1)	– Tips for young carers or siblings to take care of themselves – Tips for parents to support the siblings – Conversation tips for professionals, parents or siblings/young carers
Interviews or blogs	58 (23%)	Siblings (18); young carers (17); broad audience (15); parents (10); care professionals (3); not specified (4); teachers (1)	– Interview with young siblings about their experiences – Interview with adult siblings about childhood experiences – Blog by parent that also shares something about the siblings
**Fun activities**			
Fun activity for siblings or young carers	81 (32%)	Young carers (61); siblings (20); parents (6); not specified (1)	– Fun activity with the parent and/or sibling with a disability – Fun activity (e.g., canoeing, climbing) where siblings can also bring friends – Fun activity (e.g., baking) or fun day together with other siblings or young carers
Vacation week(end)	14 (5%)	Young carers (7); siblings (7); parents (5); children in a stressful situation (1)	– Vacation for families of a child with a disability – Yearly young carer vacation week – Sibling camp
Family day or activity	5 (2%)	Parents (5); siblings (4); young carers (1)	– Day with activities for families of a child with a disability – Barbecue for the families of the children at the care facility – Day with activities for fathers and siblings
**Interventions or support groups/persons**			
Contact person for siblings or young carers	60 (23%)	Young carers (57); parents (2); broad audience (1); personal network of a child with autism (1)	– Young carer counsellor who can be contacted for support – 24‐h phone number children can call to talk – Phone number for information about young carer support
Group intervention for siblings or young carers	56 (22%)	Siblings (40); young carers (20); parents (10); children in a stressful situation (2)	– Sibling support group with multiple sessions and a final session with parents – Boardgame to have a conversation with siblings about several themes – Programme targeted at emotions, that use playful elements (e.g., ‘Billy Boem’, ‘Piep Zei de Muis’)
Individual intervention for siblings or young carers	22 (9%)	Siblings (16); young carers (7); parents (4)	– Boardgame or computer game for siblings – Individual or parent–child coaching for siblings – Individual support from a family counsellor
Buddy project	19 (7%)	Young carers (19)	– Buddy to do weekly fun activities with – Adult buddy who was a young carer themselves to talk with – Volunteer that the young carer can do activities with or text
Peer contact (not specified)	16 (6%)	Siblings (10); young carers (6); parents (2)	– Unspecified ‘peer contact’ – Day for siblings (no details provided) – A group where you can meet other siblings (without a program or activity)
**Other**			
Gift	36 (14%)	Young carers (33); siblings (3); parents (1)	– Occasional gift (e.g., for the Week of the Young Carer) – Goodie bag or surprise box if you sign up as a young carer – Yearly gift if you are signed up as young carer (e.g., gift card or €30 cash)
Video^a^	16 (6%)	Young carers (9); professional caregivers (4); parents (3); broad audience (2); siblings (1)	– Vlog about using a support toolbox – Videos of young carers that share their experiences – Promotion video about a tool or activity
Book^a^	8 (3%)	Siblings (6); parents (2); broad audience (3); teachers (1); personal network of child with care needs (1)	– Sibling book with personal stories and tips – Book for siblings of children with Down Syndrome – Book for teachers about supporting young carers
Other	54 (21%)	Siblings (25); parents (15); care professionals (12); broad audience (11); young carers (7); families (3); teachers (1); researchers or organisations (1)	– Podcast interviews with siblings – Theatre play about a sibling story – Workshop for professionals – E‐learning for parents – Conversation cards, worksheets, ‘sibling beads’ craft pack – Respite services or guest families – Toolkit for schools about young carers – Family therapy that also includes the sibling

^a^ This only includes books and videos that are produced by the organisation itself, not references to these sources of other providers.

^b^ Multiple target groups could be selected, and thus the sum of the frequencies is not equal to the total number of (cluster) resources of one type.

^c^ ‘Brus’ is the Dutch word that is used to indicate siblings of children with care needs.

##### 3.2.1.3 Target Groups

Of all 707 (clustered) resources provided by organisations, most targeted a broad group of young carers (*n* = 376) and/or siblings of children with a broad range of diagnoses (*n* = 175). Few specifically targeted siblings of children with intellectual disabilities (*n* = 33) and/or visual impairments (*n* = 5). Some resources (also) targeted parents (*n* = 119), professional caregivers (*n* = 67), and/or a broad audience (*n* = 54). Other target groups (*n* = 36) included, for example, teachers, specific groups of siblings (e.g., of children who have autism or auditory impairments) and persons that know a family with a child with a disability. For 484 resources (68%) no specific age group was mentioned or applicable. For those that did indicate a specific age group there was high variability. Some included only a minimum age, others only a maximum age and others a range. The indicated minimum ages ranged from 4 to 12 years (*M* = 7.34; SD = 2.24).

##### 3.2.1.4 Accessibility of Resources

Most of the resources offered by organisations were free of cost (80%). Of the interventions and activities, most were on site (*n* = 202), with only some digital options (*n* = 19). Some organisations offered activities and interventions once or multiple times per year (*n* = 138) or per month (*n* = 42). Others were one‐time initiatives (*n* = 33).

##### 3.2.1.5 Books, News Articles, Research Texts or Reports and Media

Table 4 provides an overview of characteristics of the found books, news articles, research texts or reports and media sources. Of the books, only a few were available for free (12%). Most books were targeted at siblings or a broad audience, with a minimum age ranging from 3 to 12 (*M* = 6.68, SD = 2.58). In 41% of the books, the age of the target group was not indicated or not applicable. Some (*n* = 13) of the books were more than 20 years old (oldest from 1993), and some appeared outdated (e.g., stigmatising language use). Nearly all media sources, news articles and research texts or reports were targeted at a broad audience or professionals.

**TABLE 4:** Information about books, news articles, scientific texts and media sources.

Resource type	*N*	Type (*n*)	Examples
Books	65	Story/picture book (20); fictional youth book (14); informative book for parents and/or professionals (7); book with personal stories (7); activity book (6); adult book about personal experiences (5); informative book for siblings (4); comic book (2)	– Book for young siblings with tips and information about Down Syndrome – Personal stories and photos of siblings from different ages – Fictional storybook about siblings of which one has a disability – Informative book about sibling relationships and siblings of children with disabilities or other conditions
Media sources	30	Tv series or episode (7); documentary (5); YouTube video (7); podcast (3); radio or tv talk show (3); fictional film (1); Instagram page (1); educational clips (1); youth news item (1)	– Videos of young carers that share their experiences – Fictional tv series for children about a sibling of a child with an intellectual disability – Sibling that shares her (childhood) experiences in a talk show – Instagram page with small comics about difficulties that siblings experience
News articles	20	Regional newspaper or broadcaster (10); national newspaper (3); magazine (2); professional journal (1); other (4)	– Interviews with sibling or young carers about their experiences – Article that announces an activity or reports about the activity – Article about research related to siblings – Article from a psychologist about the experiences of siblings
Research texts or reports	19	Thesis or study project (9); summary of scientific work (2); report (7); poster (1)	– Thesis about the impact of having a sibling with an intellectual disability – Government report with 25 case descriptions of (young) carers – Quickscan of the support needs and support resources for siblings – Report about what works in supporting young siblings, based on a literature review

#### 3.2.2 Match Between Sibling Resources and Siblings' Needs

Table 5 provides an overview of frequencies and examples of the themes that were mentioned in the found resources. It shows that relatively few resources explicitly mentioned difficulties in relation to understanding their sibling, challenging behaviour of their sibling, or reactions from others. In addition, few resources seem to be focused on learning skills or solution‐focused help.

**TABLE 5:** Themes identified in the resources.

Themes found in interviews (Part 1)	Number of resources from organisations (*N* = 707) (Part 2)	Number of other sources (*N* = 134) (Part 2)	Examples from the resources (Part 2)^a^
**Themes about the challenges that siblings experience**			
Struggling to understand their sibling	28 (4%)	28 (21%)	– Interview: *‘I don't always understand my brother too well and then I don't know what to do. I would like to take on something of my brother, so that I would feel what he feels, because then I can also understand it better’* (Website #220: Week van de Jonge Mantelzorger) – Description of sibling course: *‘You can think of questions such as Why does my brother/sister react that way? Or Why does he or she do those things?’* (Organisation #248: Yulius)
Challenging behaviour of their sibling due to the disability	12 (2%)	20 (15%)	– Interview: *‘Senna sometimes targeted her frustration on her 4‐year‐old sister. ‘She really hated that, but she could not control it’, says Natasja. That really got out of hand, and we did not know what to do any longer. We sought a good way to handle Senna and bring back the peace in our family.’* (Organisation #211: Vereniging Gehandicaptenzorg Nederland) – Question in workbook: ‘*If he hits you, do you get angry or not?*’ (Book #59: Vragen voor en door brussen)
Adapted family interactions and activities	156 (22%)	71 (53%)	– Description of sibling toolkit: *‘Within such a family much of the attention of the parents goes to the disabled child. That is logical. But therefore, the brothers and sisters unintendedly receive less attention’* (Organisation #4: Amarant) – News article: *‘A spontaneous trip or vacation is not an option, that is too much or unclear for Caithlyn.’* (Article #16: RTV Noord)
Judgemental reactions from others	44 (6%)	35 (26%)	– Information page: *‘Sometimes other children say stupid things about your brother/sister. Of course, that does not feel so nice.’* (Organisation #107: Kind Zoekt Hulp) – Book description: *‘Why is he in a wheelchair? Why can't he walk? Is your brother broken? Why does he look so weird? And can he not say anything? Isn't it annoying to have a brother like him? Lisa gets a lot of questions from others about her brother Mikko, who has multiple disabilities. These questions show misunderstanding and bother her.’* (Book #49: Mikko mijn stoere broer)
Negative feelings in relation to their sibling's disability	159 (22%)	78 (58%)	– Description of a sibling support group: *‘Some siblings are worried about their brother or sister or about their parents. Feelings of sadness, shame and anger are also common.’* (Organisation #134: MEE Dichtbij) – Radio interview: *‘Feelings were all over the place. From guilt, to irritation, to pride.’* (Media source #30: NPO)
**Themes about the kinds of support that siblings want to receive**			
Receiving information	116 (16%)	31 (23%)	– Description of support group: *‘In this group, your child receives explanations about the disabilities […]’* (Organisation #25: Centrum voor Jeugd en Gezin Breda) – Book: *‘The book can be used to explain things that they don't understand. […] In this joyful, illustrated book, we give you information, ideas and tips.’* (Book #1: Alle vragen over Downsyndroom)
Learning new skills	71 (10%)	24 (18%)	– Video: *‘In the course I learned how to handle my brother with a disability, when he is angry, I ignore him.’* (Media #22: RTV Rijnmond) – Book: ‘*By learning children that all feelings are allowed, and making them recognisable* via *a story, they learn how to cope with these feelings*.’ (Book #26: Het is oké…)
Solution‐focused help in the moment	39 (5%)	8 (6%)	– Tips for parents: *‘Sometimes it is inevitable to ask the sibling for help; because you also need to go to the toilet or need to grab something upstairs when the school bus has not arrived yet. How can you do that in a way that burdens the sibling as little as possible? By demarking the task. Explain what exactly you need help with. How long it takes. What exactly you expect. If something happens, what then? Provide clear explanations. Thank the sibling afterwards and ask how it went. And tell that now you are responsible for their brother or sister and that the sibling can go playing again.’* (Organisation #8: Balans) – Interview/blog: *‘What I have learned from this situation is that it is good to try to find solutions together when you are facing problems.’* (Organisation #180: Sophi)
Contact with peers	165 (23%)	30 (22%)	– Description of support group: *‘You meet other youths that are in the same situation as you. You can share experiences and learn from each other's tips and tricks.’* (Organisation #54: En Nu Jij) – Description of fun activities: *‘With peers that, just like you, need to consider the needs of someone else. Just having fun, distraction and being together. No strings attached. But of course, you can also tell us your story.’* (Organisation #36: Cordaad Welzijn)
Emotional support	166 (23%)	33 (25%)	– Description of buddy project: *‘At a buddy or guest family you can blow off steam, have a shoulder to cry on, or take your mind of things.’* (Organisation #57: Evenmens) – Description of individual support: *‘It can be worthy to siblings that they can share their feelings with. Our care professionals can support the sibling and help them cope with their sibling's disability.’* (Organisation #42: De Zorgnijverij)
Receiving quality time and attention	132 (19%)	26 (19%)	– Tips for parents: *‘Regularly make extra time for the siblings’* (Organisation #221: Wegwijs) – Description of fun activity: *‘Two times a special afternoon was organised for the siblings of the children at daycare center AandachtsLab. We find it very important that the siblings also receive special attention.’* (Organisation #3: AandachtsLab)

^a^ Information about the organisations or sources that are referred to can be found in Supporting Information S4.

## 4 Discussion

In the first part of this study, the aim was to gain insight into the self‐reported support needs and preferences of siblings (6–12 years) of children with intellectual disabilities and/or visual impairments. The second part aimed to provide an overview of the support resources that are available in the Netherlands and Belgium, and to what extent these match siblings' needs.

Interviews revealed that siblings face various challenges that they would like different types of support with. The challenges they experienced were in line with those reported in previous studies and that are of importance to their quality of life (Moyson and Roeyers 2012; Múries‐Cantán et al. 2023). For example, siblings can experience challenges related to the behaviour of their sibling or the reactions from others. The conclusion that the experiences of siblings are personal and context‐specific (Múries‐Cantán et al. 2023) was reaffirmed by the variation in difficulties that siblings mentioned.

This study adds new insights into the various support needs and preferences that siblings express in relation to these challenges. This includes the need for *social and emotional support*, *solution‐focused help in the moment*, *receiving information*, *learning new skills*, *receiving quality time and attention*, and *contact with peers*. A few siblings mentioned that they do not need support with (some of) their challenges, or that these experiences have become ‘normal’ to them. This might reflect acceptance of their situation but could also show lack of awareness, or a reluctance to express their emotions or to ask for support (Hanvey et al. 2022; Haukeland et al. 2015). Again, these findings are in line with the support needs reported in previous studies (Hanvey et al. 2022; Joosten et al. 2019; Múries‐Cantán et al. 2023) among other target groups (e.g., teenagers) and using other methods (e.g., retrospective interviews). The need for solution‐focused help, such as a parent that helps to end their siblings' disruptive or aggressive behaviour, was not mentioned in these previous studies and thus provides a new insight.

Moreover, the current study adds information about whom siblings wish to receive support from. For the school‐aged siblings in this sample, the most important support persons are their parents. Previous research has stressed the importance of support from parents (Marquis et al. 2022) and family members in middle childhood, but also of teachers, as children spend a lot of time at school (Oberle et al. 2014; Steed and Langlais 2025). Notably, none of the interviewed siblings mentioned their teachers as providers of support with challenges they experience in relation to their sibling with a disability. This might indicate that they perceive their school‐life as separate from their family‐life. But it might also indicate that they do not view teachers as approachable or equipped to support them with issues related to their sibling and/or the disability. Similarly, Pavlopoulou et al. (2022) reported that siblings can be reluctant to ask teachers for support with matters related to their sibling, as teachers do not always fully understand their situation.

In the second part of this study, a grey document review of support resources for siblings in the Netherlands and Belgium revealed that various forms of support can be found online for siblings. These resources are scattered over the health and social care system, with a total of 255 organisations providing some sort of support to siblings or young carers. These resources can offer direct or indirect (e.g., information for parents or professionals) support and can be categorised in information sources, fun activities, interventions or support groups/persons, and other resources (e.g., gifts, videos). In addition, 134 books, articles, texts, reports and media sources for or about siblings or young carers were found. At first glance, it appears that many resources are available to siblings. However, most of the organisations only included general tips, basic information, references to other resources, or fun activities. In addition, most activities and interventions were only offered a few times a year or only in some municipalities. Also, in some cases it was not always clear if the interventions and activities that were mentioned on the websites were still offered, as not all websites appeared up to date (e.g., still showing activities offered in previous years). Furthermore, most of the preselected websites of care organisations that support children with disabilities did not contain any sibling support resources (90%). Finally, the quality and evidence for the effectiveness of the interventions that are offered is unclear.

Several gaps in the support offer were identified. First, as was also found in a study in the United Kingdom (Taylor et al. 2025), most of the offered resources target a broader group, namely young carers. For fun activities and gifts, this does not (always) provide a disadvantage. A drawback for other support resources is that they are not tailored to the specific needs of siblings, who can get overlooked within the broader group of children growing up in families with other care needs. Specific needs that siblings indicated in this study, such as receiving information about the disability or learning to deal with the behaviour of their sibling, are likely not addressed in these broader support groups. Moreover, siblings that participated in this study and a previous study (Joosten et al. 2019) mentioned that they wish to exchange experiences with others that experience similar things, which is not the case in heterogeneous support groups (e.g., children with parents with an addiction).

In addition, few resources aimed to train skills or provided solution‐focused support, and few addressed difficulties in understanding their sibling, coping with others' reactions, and managing their siblings' challenging behaviour. Especially the latter is important, as research shows that behavioural problems of the child with a disability are a risk factor for developing psychosocial problems in siblings (Wolff et al. 2022). Moreover, during the interviews, some siblings explicitly expressed the need to learn how to manage aggressive behaviour. This highlights a significant gap in existing sibling support resources. These unmet support needs might increase the risk of developing mental health problems (Wolff et al. 2025) and call for improvements in sibling support.

### 4.1 Strengths and Limitations

This study includes several strengths and limitations. It is the first to focus on self‐reported support needs of young siblings of children with disabilities, which is a strength, as it provides first‐hand information. The use of visual prompts in the interviews helped to structure the interview and enable the communication (Glegg 2019). Also, it was a strength that the interviews were conducted in the absence of the parents, as some siblings can be reluctant to share difficulties in the presence of their parents out of loyalty and protection (Houtzager et al. 1999). However, a limitation was that siblings were only interviewed once, providing limited time to build a relationship with the interviewer or to expand their insights on their own needs. Repeated interviews could have provided deeper and more nuanced insights (Goyes and Sandberg 2025). Finally, only two siblings had a brother or sister with a visual impairment without an intellectual disability, of which only one sibling had a brother or sister with a visual impairment without an additional (diagnosed) developmental disability. Although no other themes arose from the interviews with these siblings specifically, it could be that not all the themes mentioned in the total sample also apply to all siblings of children with visual impairments. Thus, further research into the needs of this specific group might be needed.

Regarding the grey document review, a strength was that an overview of the sibling support in the Netherlands and Belgium is provided that can be used to guide practice. Although the results are not generalizable to other countries, they do provide insights about sibling support that could inspire researchers and practitioners. For example, researchers could map the resources that are available in practice internationally and exchange best practices. However, this study only provides an overview of resources that can be found online. Organisations might offer other supports that are not presented on their websites. Surveys could provide additional information on the support that is offered.

Another limitation was the complexity in the data extraction, as it was sometimes hard to categorise the information. For example, the organisation type was not always clear, and the resource types sometimes overlapped (e.g., contact with peers and group interventions). Also, the researchers were all Dutch and therefore less familiar with the Belgian care system. Future studies are recommended to include researchers with local expertise to improve the comprehensiveness of the findings.

Finally, this study only assessed if the themes from the interviews are represented in the texts of the found resources but does not indicate to what extent the resources provide support with these themes. For example, themes are applied to examples that are given by siblings in interviews or blogs, but that does not mean that the interview provides support with this specific challenge. Still, this study provides information on the themes that are underrepresented in the resources.

### 4.2 Implications for Research and Practice

This study provides several implications that can guide practice, policy and research in improving sibling support. First, it is advised that the existing support resources are made better findable and accessible, for example on a central website. Herein, it is important to ensure that information and resources on organisations' websites are up‐to‐date and accessible. In addition, awareness about the possibility to use young carer resources needs to be raised, since most families, care professionals and teachers are not aware that siblings are young carers (Smyth et al. 2011).

Second, future studies could further assess the theoretical and empirical background, as well as the quality of the interventions that are offered in practice. In addition, it would be valuable to assess to what extent evidence‐based interventions that are presented in scientific papers (e.g., Kirchhofer et al. 2025) are available in practice.

Third, the identified gaps in sibling support need to be addressed. Organisations are advised to provide more targeted support specifically for siblings, tailored to their unique experiences. In addition, more resources need to be offered that provide skills training and practical solutions, especially for handling aggressive behaviour. In this matter, combining support with enjoyable activities can keep siblings engaged and make support more approachable. Moreover, a personalised approach in supporting siblings is advised, as their needs and preferences are diverse (Múries‐Cantán et al. 2023). The results from this study could be used to develop a comprehensible ‘support menu’ that siblings can use to indicate their support needs and preferences. Future research could focus on developing an instrument to assess the support needs of siblings, such as was developed for siblings of children with cancer (Patterson et al. 2011).

Fourth, parents should be involved as key support figures. Siblings, like all children, first and foremost need parents that are sensitive and responsive to their needs (Santana‐Ferrándiz et al. 2025). Parents can offer a *secure base* from which siblings can learn new skills, and a *safe haven* that they can return to for comfort and safety, for example when they are frightened of their sibling's behaviour (Hoffman et al. 2006). When needed, professionals can play a role in strengthening parents' ability to support siblings effectively. Future research could focus on gaining insight into the needs that parents have in relation to providing support to siblings. Parents might for example wish for support in communicating with their children about the disability or their emotions (Schumann et al. 2024). However, not all responsibility should be placed on the shoulders of the parents, who already experience high care burdens (Patty et al. 2024). Schools and disability care organisations could play a larger role in supporting siblings. Teachers and care professionals should be more approachable and equipped to support siblings. For example, the Young Carer Friendly School project *(Jonge Mantelzorg Vriendelijke School)* (Stichting JMZ Pro n.d.), that provides a toolkit for schools about young carers, could be extended with specific components for siblings, such as the sibling school intervention that was developed in the United Kingdom (Hayden et al. 2019). In addition, teachers can play an important role in referring siblings to the resources that are offered by social workers.

### 4.3 Conclusion

The current study provides valuable insights into the self‐reported support needs and preferences of school‐aged siblings. It stresses the importance of considering the diverse needs that siblings may have, and to help them better express their needs. Moreover, it provides an overview of the available sibling support resources in the Netherlands and Belgium that could guide sibling support policies. Improving sibling support requires making services more visible, better coordinated, and focused on the needs of the whole family, rather than fragmented across different providers. Additional skills training, especially for dealing with sibling aggression, is needed. Finally, this study provides examples of sibling support that could guide organisations that do not yet offer support to siblings.

## Author Contributions

**Linda K. M. Veerman:** conceptualization, data curation, formal analysis, investigation, methodology, project administration, visualisation, writing – original draft. **Agnes M. Willemen:** conceptualization, methodology, supervision, validation, writing – review and editing. **Suzanne D. M. Derks:** conceptualization, methodology, writing – review and editing. **Anjet A. J. Brouwer‐van Dijken:** conceptualization, validation, writing – review and editing. **Paula S. Sterkenburg:** conceptualization, funding acquisition, methodology, supervision, validation, writing – review and editing.

## Funding

This work was supported by The Netherlands Organisation for Health Research and Development ZonMw, Den Haag, the Netherlands, as part of the Academic Collaborative Center ‘Affect‐us’ (grant number 641001101). The funding source did not have any involvement in this study.

## Ethics Statement

Ethical approval was obtained from the Ethics Committee of the Faculty of Behavioural and Movement Sciences of the Vrije Universiteit Amsterdam (VCWE‐2022‐173).

## Consent

Both parents of the participants and the children aged 12 years provided written informed consent. Explicit consent was obtained for publishing quotes. All children provided assent to being interviewed.

## Conflicts of Interest

The authors have developed a sibling intervention themselves, the serious game Broodles (Veerman et al. 2025), which was also included in the current grey document review. However, the intervention is free of cost and will thus not provide the authors with financial gains. In addition, Anjet Brouwer‐van Dijken was involved in several support resources that are included in the review. Although most of these resources are available free of cost, she has (very limited) financial gain from sold book copies. The authors declare no other conflicts of interest.

## Supporting information


**Supporting Information: S1.** Visual aids A–C that were used in the interviews.

**Supporting Information: S2.** List of searched websites (Table S2.1).

**Supporting Information: S3.** Extraction form support resources for siblings.

**Supporting Information: S4.** List of included resources (Tables S4.1–S4.5).

## Data Availability

The qualitative data from the interviews are not available, to protect the privacy of the participants, and because no consent was provided for sharing the data. An overview of the found resources is provided in Supporting Information S4. This includes data from resources that are available in the public domain. The complete data files of the review are available from the corresponding author, Linda Veerman, upon reasonable request.
