# Supplementary material for: Support Needs and Available Resources for School‐Aged Siblings of Children With Disabilities: A Mixed Methods Study
Source: J Appl Res Intellect Disabil. 2026 Jan 30;39(1):e70190. doi: 10.1111/jar.70190 (PMC12856529; doi:10.1111/jar.70190)

Persons that are important to me

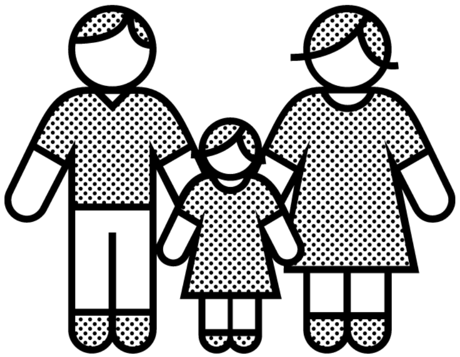

My parents

……………………..   ………………………..

………………………   ………………………

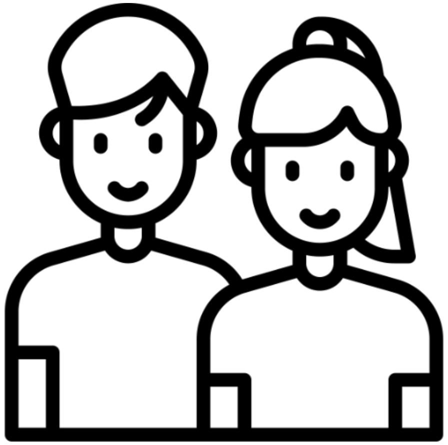

My siblings

……………………..   ……………………..

………………………   ……………………..

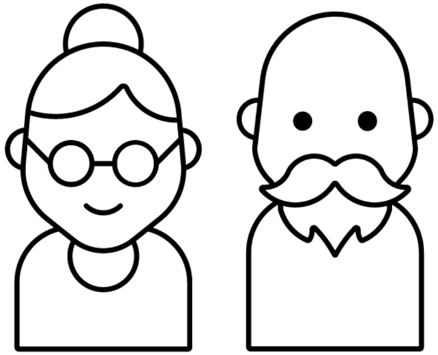

My grandparents

……………………. ……………………..

……………………… ……………………..

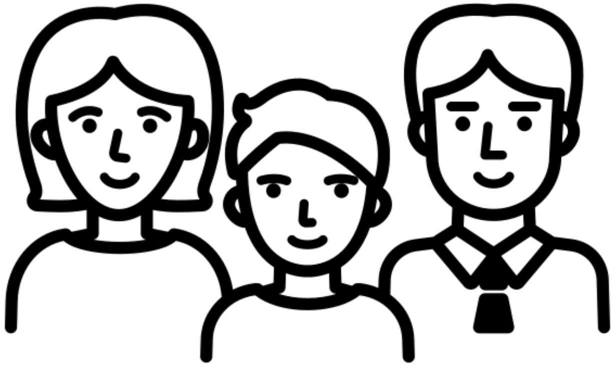

My other family members

……………………. ……………………..

……………………… ……………………..

……………………… ………………………

Supporting Information S1. **Visual Aid A**

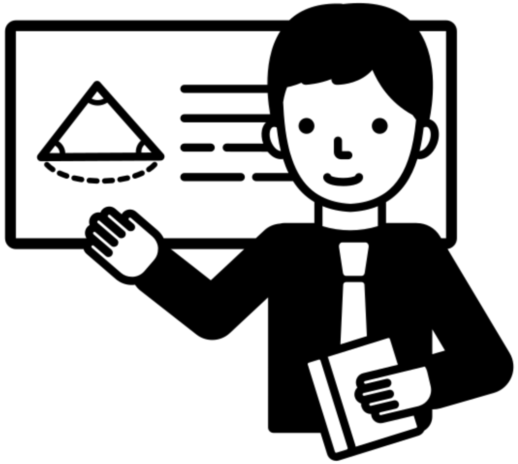

My teacher

……………………..

……………………….

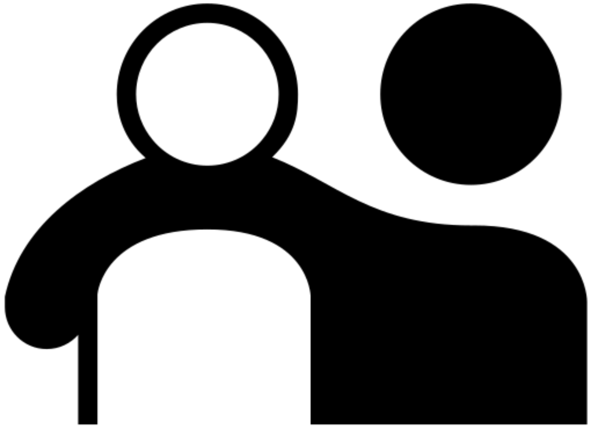

My friends

…………………….. ……………………..

………………………. ……………………..

………………………. ……………………….

Supporting Information S1. **Visual Aid A**

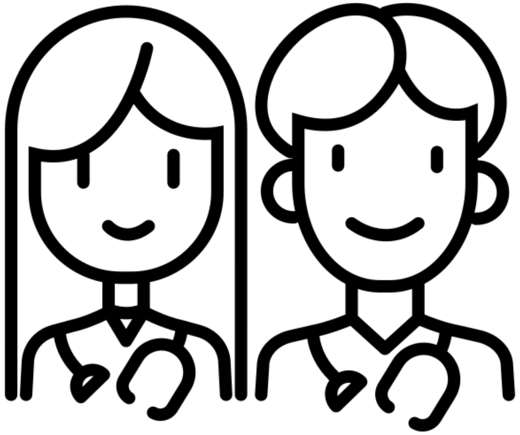

Persons in the hospital

……………………..   ……………………….

……………………….   ……………………….

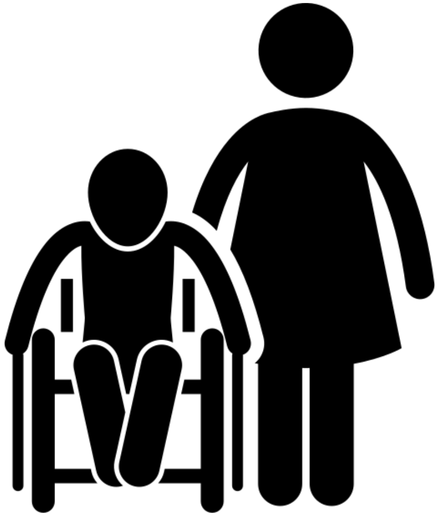

Caregivers

……………………..   ……………………….

……………………….   ……………………….

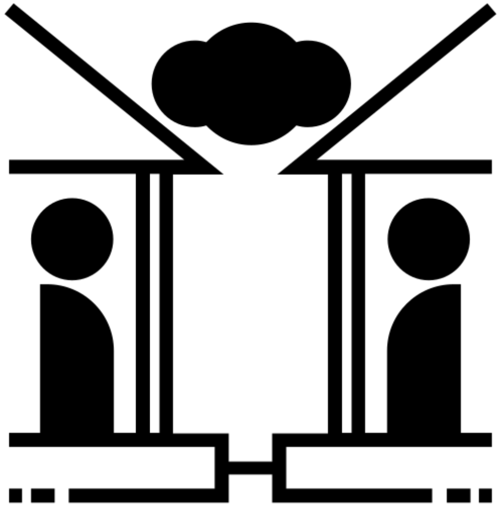

My neighbours

……………………..   ………………………

………………………   ………………………

………………………   ………………………

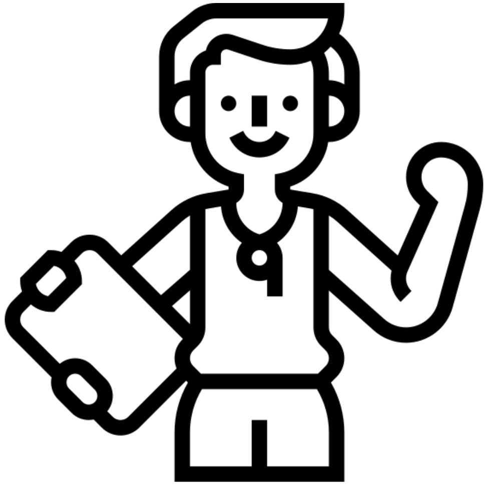

Persons at my sports club or scouting

……………………..   ………………………

………………………   ………………………

Supporting Information S1. **Visual Aid A**

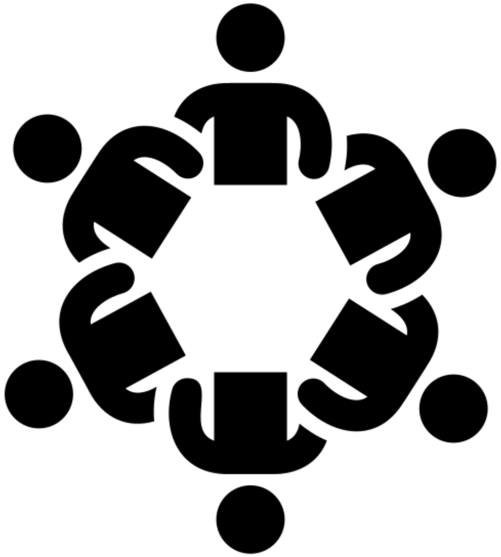

Sibling group

…………………….. ……………………..

…………………….. ……………………..

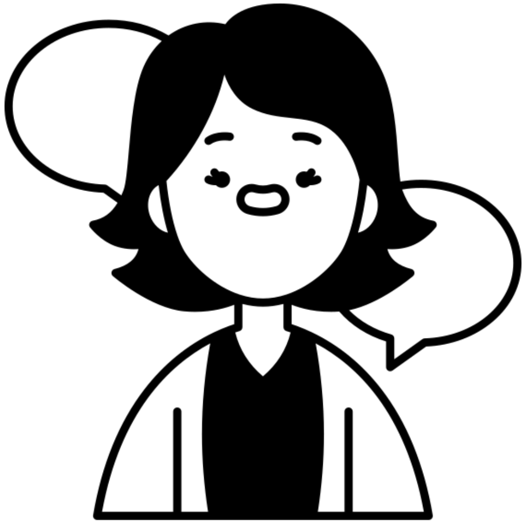

My psychologist or child coach

……………………..

……………………..

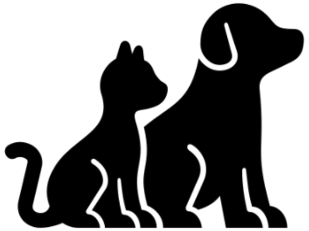

My pets

……………………..   ………………………

………………………   ………………………

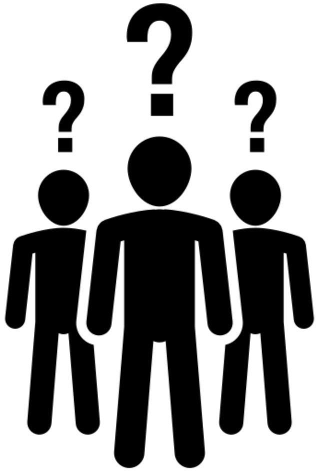

Other persons

……………………..   ………………………

………………………   ………………………

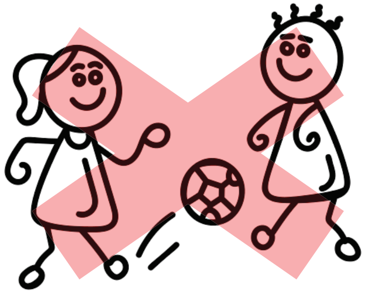

Not being able to do things together

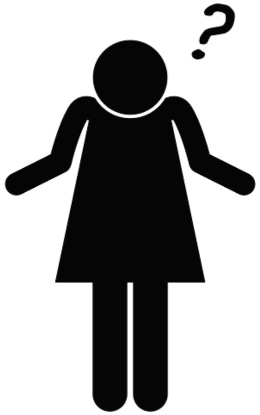

Not always understanding each other

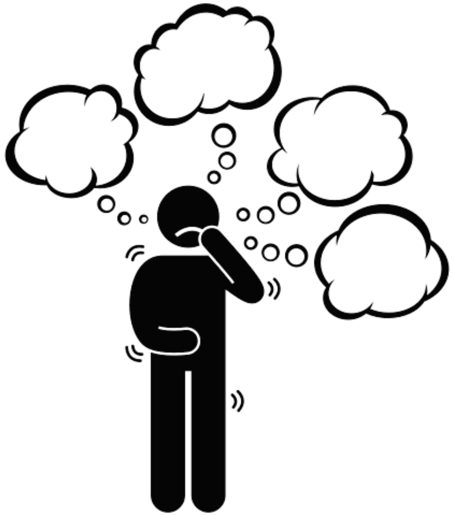

Worrying

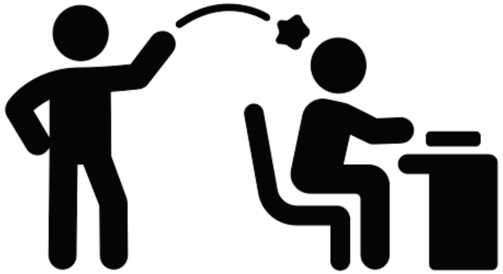

Being disturbed / not having time for myself

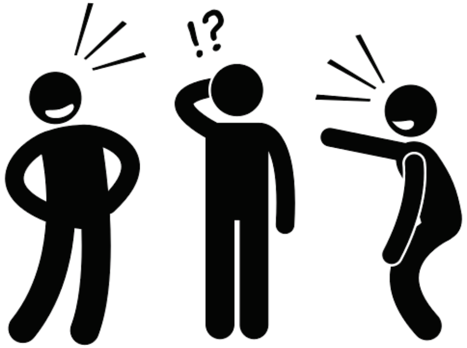

Strange reactions from others

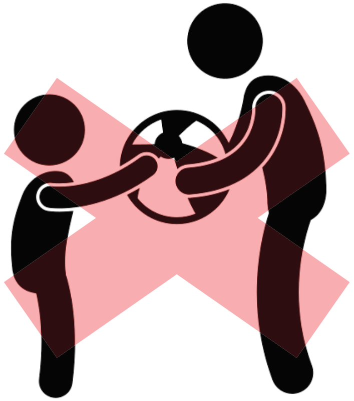

My parents have little time for me

Supporting Information S1, **Visual aid B**

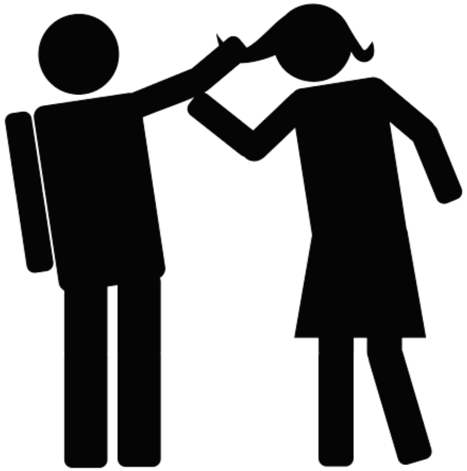

My sibling hurts me

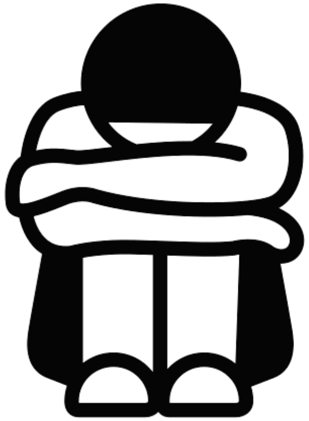

Not feeling good

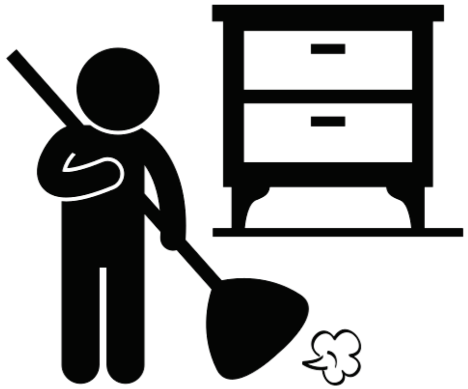

Having to help more

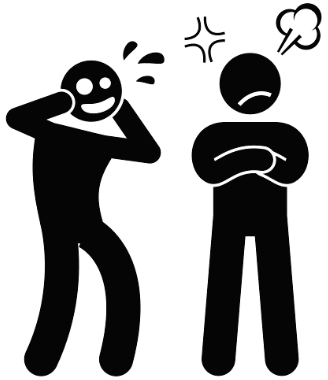

My sibling acts strange or annoying

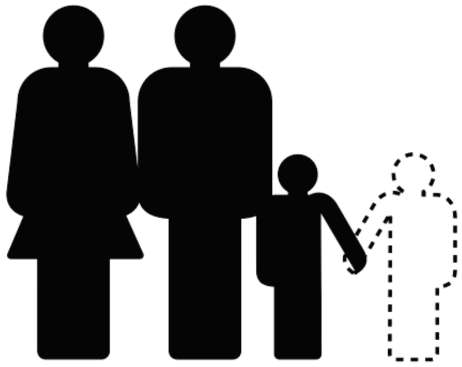

Feeling invisible

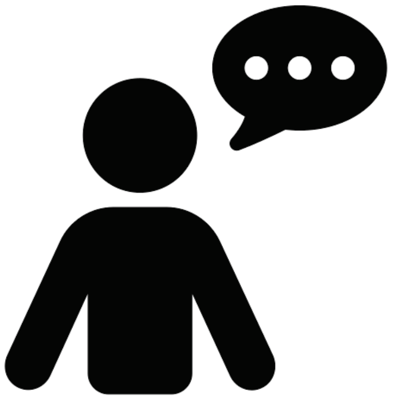

Something else

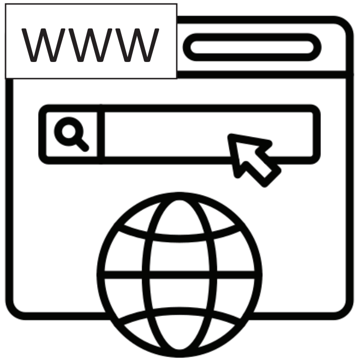

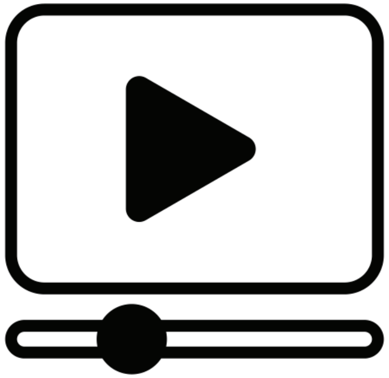

GET IN ON
Google Play

Download on the
App Store

Getting information

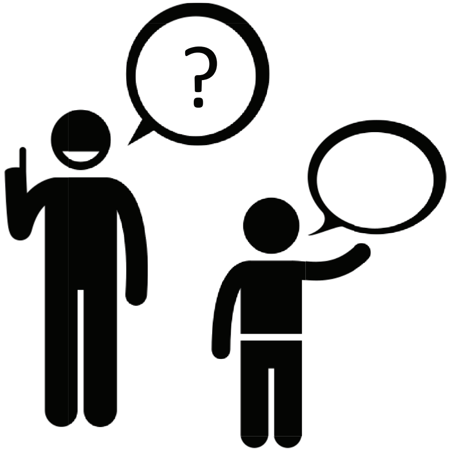

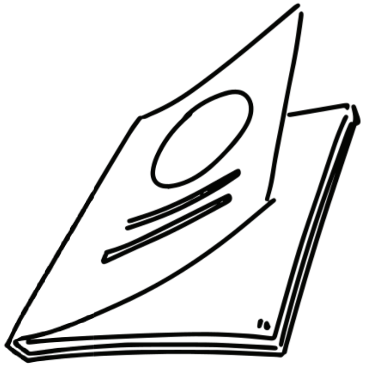

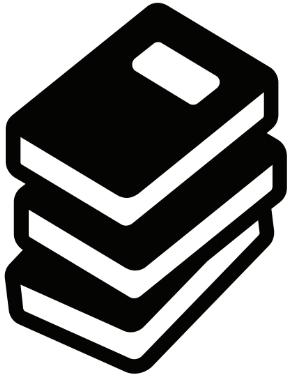

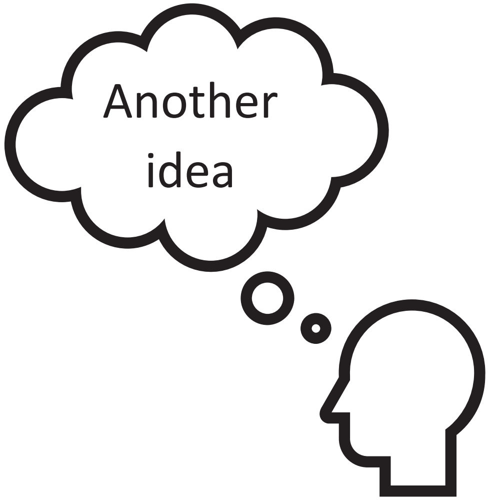

Supporting Information S1, **Visual aid C**

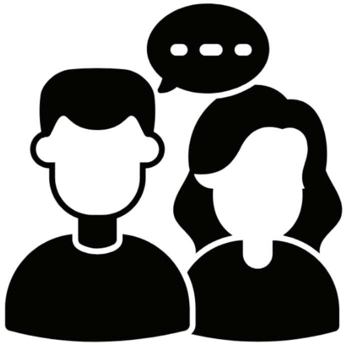

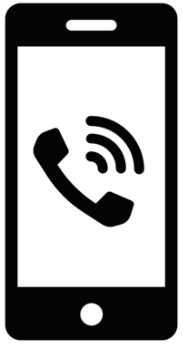

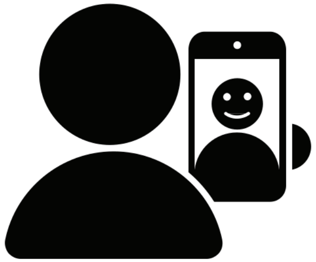

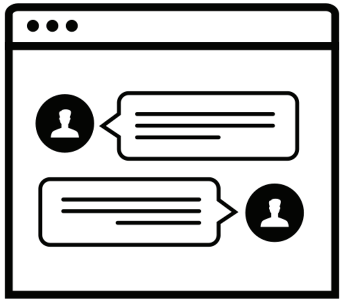

Talking about it

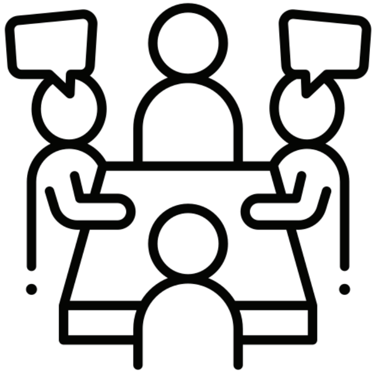

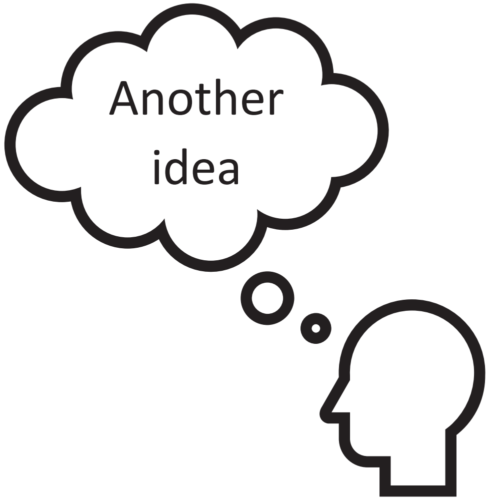

Supporting Information S1, **Visual aid C**

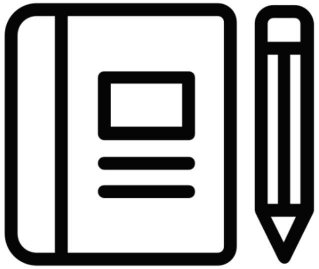

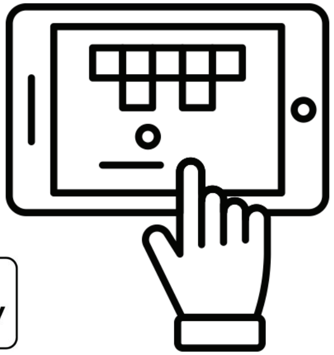

GET IN ON Google Play

Download on the App Store

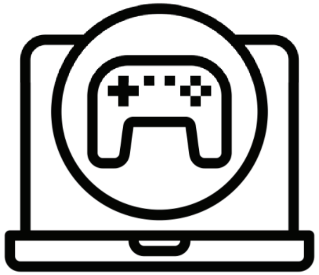

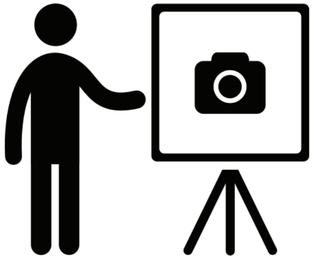

Learning how to deal with it

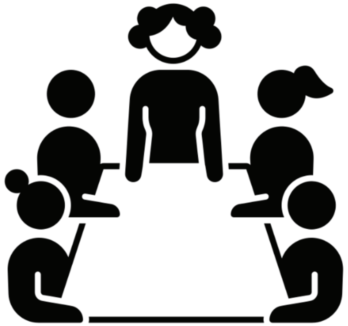

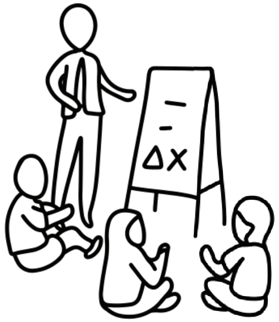

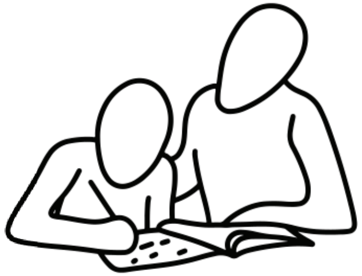

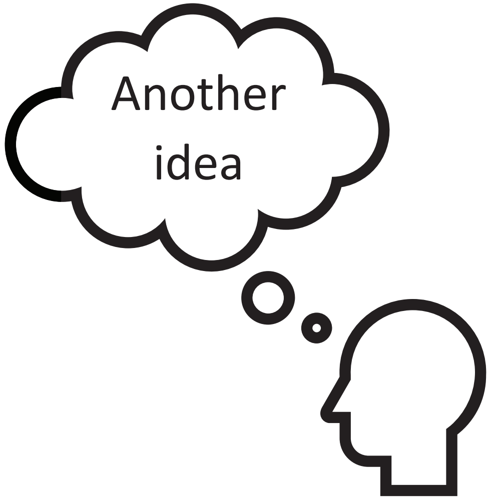

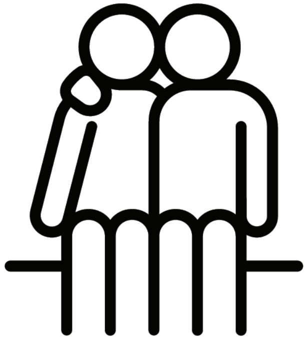

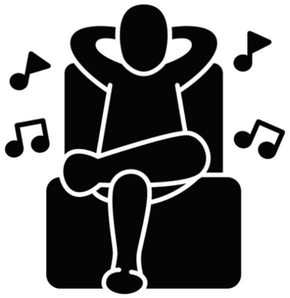

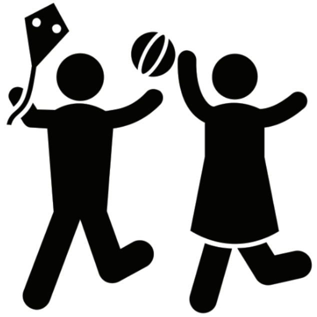

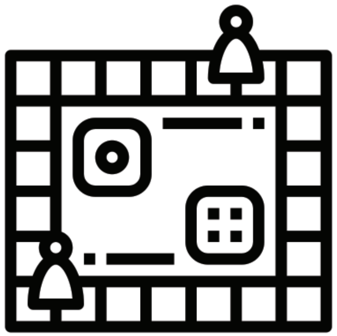

Something nice especially for me

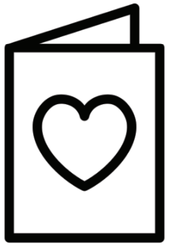

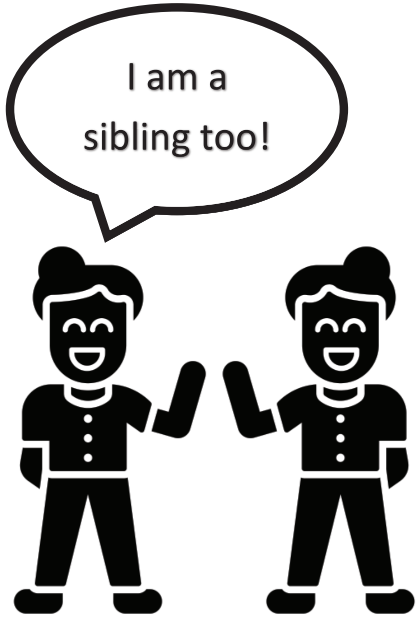

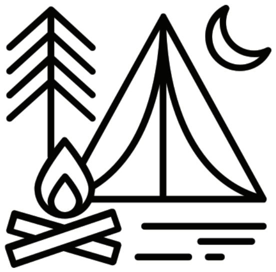

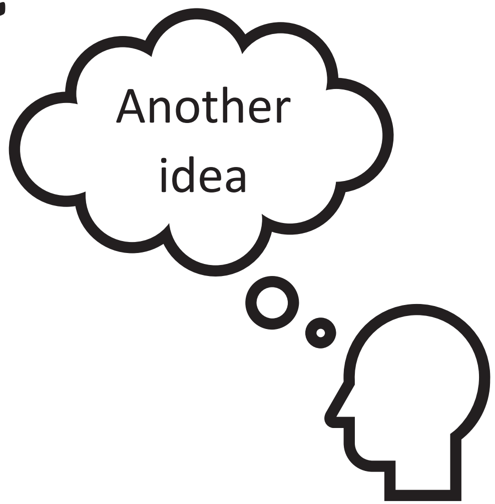

Something else

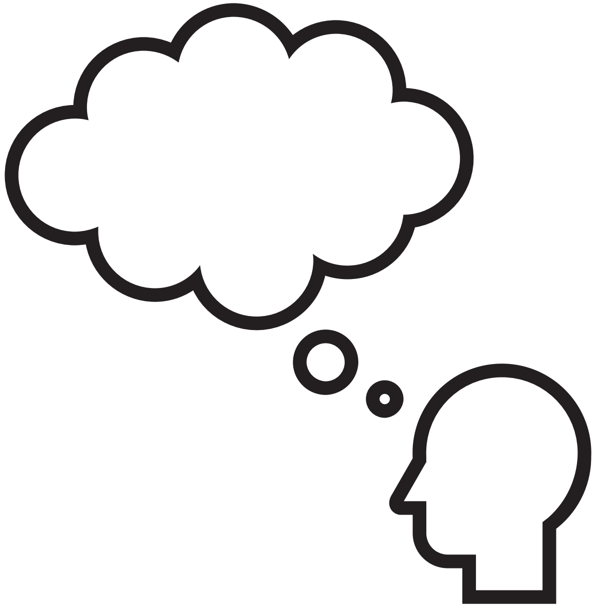

Supplement: Supplementary file 1 — Supporting Information: S1. Visual aids A–C that were used in the interviews. [file JAR-39-e70190-s001.pdf]
