# Supplementary material for: Support Needs and Available Resources for School‐Aged Siblings of Children With Disabilities: A Mixed Methods Study
Source: J Appl Res Intellect Disabil. 2026 Jan 30;39(1):e70190. doi: 10.1111/jar.70190 (PMC12856529; doi:10.1111/jar.70190)
Supplement: Supplementary file 2 — Supporting Information: S2. List of searched websites (Table S2.1). [file JAR-39-e70190-s002.pdf]

Supporting Information S2. List of Searched Websites

Table S2.1

Searched Grey Literature Databases and Dutch Webpages of Organizations that Produce Grey Literature

Original name	Translation/description	URL
HBO Kennisbank	Applied Sciences Knowledge Base	https://hbo-kennisbank.nl/index
Trimbos Instituut	independent research institute on mental health and substance use	https://www.trimbos.nl/
ZonMw	Care Research Netherlands and Medical Sciences	https://www.zonmw.nl/
Rijksoverheid	Government of the Netherlands	https://www.rijksoverheid.nl/documenten
Nivel, Nederlands Instituut voor Onderzoek van de Gezondheidszorg	Dutch Institute for Research on Healthcare	https://www.nivel.nl/
Kenniscentrum Sport & Bewegen	Knowledge Center Sport and Movement	https://www.kenniscentrumsportenbewegen.nl/
Kenniscentrum LVB	Knowledge Center Mild Intellectual Disability	https://www.kenniscentrumlvb.nl/
Vereniging Gehandicaptenzorg Nederland	Association Disability Care Netherlands	https://www.vgn.nl/
Kenniscentrum Welzijn, Wonen en Zorg	Knowledge Center Well-being, Residence and Care	https://www.kenniscentrumwwz.be/
Nederlands Jeugd Instituut	Dutch Youth Institute	https://www.nji.nl/
Kennisplein Gehandicaptensector	Knowledge Square Disability Sector	https://www.kennispleingehandicaptensector.nl/
OCLC Nederlandse Centrale Catalogus	Netherlands Central Catalogue	https://picarta.on.worldcat.org/discovery
Klik Kenniscentrum Verstandelijk Gehandicaptenzorg	Knowledge Center Intellectual Disability Care	https://www.klik.org/
