# Supplementary material for: Support Needs and Available Resources for School‐Aged Siblings of Children With Disabilities: A Mixed Methods Study
Source: J Appl Res Intellect Disabil. 2026 Jan 30;39(1):e70190. doi: 10.1111/jar.70190 (PMC12856529; doi:10.1111/jar.70190)
Supplement: Supplementary file 3 — Supporting Information: S3. Extraction form support resources for siblings. [file JAR-39-e70190-s003.pdf]

Supporting Information S3. Extraction Form Support Resources for Siblings

Note. Boxes indicate that multiple options could be selected. Circles indicate that one option could be selected.

Name research assistant

____

Information about the organization/source

Name of the organization/source

____

Organization type

- ☐ Disability care organization
- ☐ (Young) carer organization
- ☐ Social youth care (municipality)
- ☐ Rehabilitation center or hospital
- ☐ Specialized youth care organization
- ☐ Private source or initiative
- ☐ Knowledge organization
- ☐ Patient or user organization
- ☐ Other, namely____

URL organization/source

____

Country of the organization/source

- o Belgium
- o Netherlands

Remarks about the organization/source *(optional)*

____

____

What types of support does the organization/source offer?

- Tips for or about siblings
- Reference(s) to other organizations or resources (e.g., a list of links or books)
- Information about siblings (e.g., what it means to be a sibling; that you are not the only one)
- Personal narrative (e.g., a blog or interview)
- Peer contact (contact with other siblings or family members to exchange experiences)
- Fun activity for siblings (e.g., a trip; not necessarily with other siblings)
- Fun activity together with other siblings
- Family day/activity
- Vacation week or weekend for families or siblings
- Group intervention for siblings (e.g., a training, workshop sibling group)
- Individual intervention for siblings (e.g., coaching)
- Buddy project
- Contact person for siblings (e.g., 'young carer consultant')
- Gift for siblings
- Book
- Podcast
- Video
- Other, namely ____

Information about each type of support resource
You will now fill out information about each type of support resource offered by this organization. Cluster the resources per support type. For example, if there are multiple pages with information about siblings cluster these as one resource. This does not account for interventions, you fill out all these as separate resources.

Support resource 1 *(*the following part of the form repeats for each type of support resource)*

Type of support resource

- ☐ Tips for or about siblings
- ☐ Reference(s) to other organizations or resources (e.g., a list of links or books)
- ☐ Information about siblings (e.g., what it means to be a sibling; that you are not the only one)
- ☐ Personal narrative (e.g., a blog or interview)
- ☐ Peer contact (contact with other siblings or family members to exchange experiences)
- ☐ Fun activity for siblings (e.g., a trip; not necessarily with other siblings)
- ☐ Fun activity together with other siblings
- ☐ Family day/activity
- ☐ Vacation week or weekend for families or siblings
- ☐ Group intervention for siblings (e.g., a training, workshop sibling group)
- ☐ Individual intervention for siblings (e.g., coaching)
- ☐ Buddy project
- ☐ Contact person for siblings (e.g., ‘young carer consultant’)
- ☐ Gift for siblings
- ☐ Book
- ☐ Podcast
- ☐ Video
- ☐ Other, namely ____

Example of the support resources that are offered of this type (e.g., example of an activity or gift). *When clustering multiple resources: descibe at least two examples*

____

____

About the target group

Target group

- ☐ Siblings of children with care needs
- ☐ Siblings of children with visual impairments
- ☐ Siblings of children with intellectual disabilities
- ☐ Young carers (general)
- ☐ Parents
- ☐ Professional caregivers
- ☐ Broad audience
- ☐ Not specified
- ☐ Other, namely ____

Age of the target group

- o Not specified
- o Specified, namely ____

Additional information about the target group *(optional)*

____

Practical information about the support resource

Costs

- o Free
- o Paid for
- o Some free, some paid for
- o Unclear

Location *(only for interventions or activities)*

- ☐ Digital/online
- ☐ At one location
- ☐ At multiple locations

Frequency *(only for interventions or activities)*

- o One time offer
- o Multiple times/activities per week
- o Multiple times/activities per month
- o Multiple times/activities per year

Number of sessions *(only for interventions or activities)*

____

Content of the support resource

Themes represented in the resource
See descriptions of themes. When no themes are applicable, leave blank and indicate this in the remarks. Only select themes when these are explicitly mentioned in the resource, do not speculate.

Focuses on these experienced difficulties

- ☐ Difficulties in understanding their sibling (e.g., not understanding their behaviour; having difficulties in communicating with one another)
- ☐ Challenging behaviour (e.g., disturbing behaviour; being hurt)
- ☐ Difficulties related to family interactions and activities (e.g., not being able to do things together, receiving less attention; having to help more; adjustments)
- ☐ Reactions from others (e.g., lack of understanding; unpleasant reactions from others)
- ☐ Emotions in relation to their sibling's disability (e.g., anger, irritation, sadness, worry, shame)

Includes this kind of help/support

- ☐ Receiving information (e.g., information about the disability; explanations about behaviour)
- ☐ Learning skills (e.g., learning how to deal with something; learning sign language)
- ☐ Solution-focused help in the moment (e.g., parents stop the behaviour; practical solutions)
- ☐ Contact with peers (e.g., exchanging experiences, an activity with peers)
- ☐ Emotional support (e.g., talking about it; being comforted)
- ☐ Receiving special attention (e.g., 1-on-1 attention from the parent; special activity or privilege)

Other themes and citations with each selected theme

____

____

____

____

Does this organization offer another type of support?

- o Yes *(fill out the form about the other types starting at *)*
- o No *(end of form)*
