# Supplementary material for: Support Needs and Available Resources for School‐Aged Siblings of Children With Disabilities: A Mixed Methods Study
Source: J Appl Res Intellect Disabil. 2026 Jan 30;39(1):e70190. doi: 10.1111/jar.70190 (PMC12856529; doi:10.1111/jar.70190)
Supplement: Supplementary file 4 — Supporting Information: S4. List of included resources (Tables S4.1–S4.5). [file JAR-39-e70190-s004.pdf]

Supporting Information S4. Included Resources

Table S4.1

Overview of Included Organizations and Websites (in alphabetical order)

#	Organization name	Organization type	URL	Types of resources
1	2BeCool	(Young) carer organization	https://2becool.nl/	Tips, references, information, fun activities, group intervention, contact person, gifts, training for professionals
2	2CU	Patient or user organization	https://www.2cu.nu/	References, blogs/interviews, peer contact, family days, vacations
3	AandachtsLab	Disability care	https://www.aandachtslab.nl	Fun activities
4	Amarant	Disability care	https://www.amarant.nl/	References, information, blogs/interviews, fun activities, videos, toolkit
5	Amerpoort	Disability care	https://www.amerpoort.nl/	References, blogs/interviews
6	Avant Sanare	Specialized youth care	https://avantsanare.nl/	References, information, group intervention
7	Baalderborg	Disability care	https://www.baalderborg.nl/	Respite care so the parent has time for the siblings
8	Balans	Patient or user organization	https://balansdigitaal.nl/	Tips, references, information, blogs/interviews
9	Bartiméus	Disability care	https://bartimeus.nl/	Group intervention
10	Basalt	Rehabilitation center or hospital	https://www.basaltrevalidatie.nl/	Child advisory board that includes siblings
11	Bijbels opvoeden	Private initiative	https://www.bijbelsopvoeden.nl/	Information
12	Bindkracht VHL	Social services	https://www.bindkrachtvhl.nl/	Tips, references, group intervention, contact person, gifts
13	Blijdorp	Disability care	https://blijdorp.be/	Fun activities, attention from family caregiver

14	Blik op hulp	Knowledge organization	https://blikophulp.nl/	Information
15	Breda Mantelzorg	(Young) carer organization	https://www.bredamantelzorg.nl/	Fun activities, contact person
16	Broeders van Liefde: Onderwijs en Zorg	Broader care and education organization	https://broedersvanliefde.be/	Information, peer contact, fun activities, experience afternoon at the care facility, sibling welcome booklet
17	BRUS	Private initiative	https://www.brussenbeweging.nl/	References, interviews/blogs
18	Brussen	Webpage of a research project (HoGent)	https://www.broersenzussen.be/	Tips, references, interviews/blogs
19	Brussengame	Webpage of a research project (VU Amsterdam)	https://www.brussengame.nl/	References, information, individual intervention (game)
20	Brussenkralen	Private initiative	https://www.brussenkralen.nl/	Craft package for a parent-sibling activity (making ‘sibling beads’)
21	Buurtgezinnen	Private initiative	https://www.buurtgezinnen.nl/	Families in the neighbourhood that help each other out
22	BWI Woensdrecht	Social services	https://bwiwoensdrecht.nl/	References, fun activities, contact person
23	Caleidoscoop Heereveen	Social services	https://caleidoscoopheerenveen.nl/	Fun activities
24	Casa Clara	Private initiative	https://casaclara.be/	Fun activities
25	Centrum voor Jeugd en Gezin Breda	Youth Social services	https://www.cjgbreda.nl/	Tips, information, group intervention
26	Centrum voor Jeugd en Gezin Capelle aan den IJssel	Youth Social services	https://www.cjgcapelleaandenijssel.nl/	Tips, individual intervention, contact person
27	Centrum voor Jeugd en Gezin Elburg	Youth Social services	https://www.cjgelburg.nl/	Tips, references, information

28	Centrum voor Jeugd en Gezin Epe	Youth Social services	https://www.cjgepe.nl/	Group intervention
29	Centrum voor Jeugd en Gezin Hollands Midden	Youth Social services	https://www.cjghm.nl/	Peer contact
30	Centrum voor Jeugd en Gezin Leiden	Youth Social services	https://www.cjgleiden.nl/	Peer contact, group intervention, podcast
31	Centrum voor Jeugd en Gezin Moerdijk	Youth Social services	https://www.cjgmoerdijk.nl/	Tips, references, information, group intervention
32	Centrum voor Jeugd en Gezin Oldebroek	Youth Social services	https://www.cjgoldebroek.nl/	Tips, group intervention
33	Centrum voor Jeugd en Gezin Rijnmond	Youth Social services	https://cjgrijnmond.nl/	Tips, references, group intervention
34	Connexa	Social services	https://connexa.nl/	Contact person, gifts
35	Coponcho	(Young) carer organization	https://www.coponcho.be/	References, blogs/interviews
36	Cordaad Welzijn	Social services	https://www.cordaadwelzijn.nl/	Tips, references, information, fun activities, group intervention, buddy project, contact person, gifts
37	De Boei	Social services	https://deboeibunschoten.nl/	Information, peer contact, fun activities, group intervention, contact person, gifts
38	De Hoogstraat	Rehabilitation center or hospital	https://www.dehoogstraat.nl/	Gifts
39	De Kap	Social services	https://dekap.nl/	Tips, references, information, fun activity, buddy project, contact person, gifts
40	De Ouders	Patient or user organization	https://www.deouders.be/	References

41	De Wereld van Anna-Sophie	Private initiative	https://dewereldvanannasophie.nl/	Letter from a mother to a sibling
42	De Zorgnijverij	Disability care	https://www.zorgnijverij.nl/	Individual intervention
43	Dealen met Down	Private initiative	https://dealenmetdown.wordpress.com/	References, blogs/interviews
44	Delft voor elkaar	Social services	https://www.delftvoorelkaar.nl/	Group intervention
45	Diagnose mantelzorg	(Young) carer organization	https://www.diagnosemantelzorg.nl/	Video
46	Distinto	Youth Social services	https://distinto.nl/	Group intervention
47	Dit Koningskind	Disability care	https://www.ditkoningskind.nl/	Interviews/blogs, peer contact
48	Dock	Social services	https://www.dock.nl/	Information, coaching cards
49	Driestroom	Disability care	https://driestroom.nl/	Interviews/blogs
50	E-learning Informele Zorg	Knowledge organization	https://www.e-learninginformelezorg.nl/	References, e-learning modules
51	Een Hart voor Limburg	Fund	https://www.eenhartvoorlimburg.be/	Financial support for sibling projects
52	Eleos	Specialized youth care	https://www.eleos.nl/	Information, fun activities, group intervention
53	Elver	Disability care	https://www.elver.nl/	Blogs/interviews
54	En Nu Jij	Youth Social services	https://ennujij.nl/	Group intervention, individual intervention
55	Equivalencia	Care farm	https://www.equivalencia.be/	Therapy for the child with a disability and the sibling
56	EstherVandaag	Private initiative	https://esthervandaag.wordpress.com/	Blogs/interviews
57	Evenmens	Social services	https://evenmens.nl/	Tips, fun activities, group intervention, buddy project, contact person
58	Facet Mantelzorg	(Young) carer organization	https://www.facetmantelzorg.nl/	Tips, references, blogs/interviews, contact person, gifts

59	Farent	Social services	https://www.farent.nl/	Tips, references, information, conversation cards
60	Fiola	Disability care	https://fiolavzw.be/	Fun activities
61	Fonds Kind & Handicap	Patient or user organization	https://www.fondskindenhandicap.nl/	Blogs/interviews
62	Forte Welzijn	Social services	https://www.fortewelzijn.nl/	Fun activities, contact person
63	Frion Zorg	Disability care	https://frionzorg.nl/	References, podcast
64	FunCare4Kids	Charity that organizes activities	https://funcare4kids.nl/	Fun activities
65	Ganspoel	Disability care	https://www.ganspoel.be/	Individual intervention
66	Gemeente Opmeer	Youth Social services	https://www.opmeer.nl/	References, blogs/interviews, gifts
67	Gezin en Handicap	Patient or user organization	https://www.gezinenhandicap.be/	Tips, references, information
68	GGNet	Specialized youth care	https://ggnet.nl/	Information, group intervention
69	Gro-up	Youth Social services	https://www.gro-up.nl/	Tips, buddy project, contact person, gifts
70	Handje Helpen	Volunteer organization	https://www.handjehelpen.nl/	Buddy project, video
71	Hartekamp Groep	Disability care	https://hartekampgroep.nl/	References, fun activities, family counselling that explicitly includes the siblings
72	Harteraad	Patient or user organization	https://harteraad.nl/	Conversation starter game, worksheets
73	Heliomare	Rehabilitation center or hospital	https://www.heliomare.nl/	Videos, family intervention that explicitly includes the siblings
74	Helpende Handen	Patient or user organization	https://www.helpendehanden.nl/	References

75	Heppie	Charity that organizes vacations	https://heppie.info/	Vacations
76	Hersenletselnet Overijssel	Rehabilitation center or hospital	https://hersenletselnetoverijssel.nl	Tips
77	Het Balanske	Family activities center	https://www.balanske.be/	Fun activities, vacations
78	Het Kindercoachhuis	Coach	https://hetkindercoachhuis.nl/	Information, individual intervention
79	Het Ronald McDonald Kinderfonds	Fund	https://www.kinderfonds.nl/	Blogs/interviews, vacation homes
80	Het Venster Nunspeet	Social services	https://www.hetvenster-nunspeet.nl/	References, guest lessons
81	Het Vlaams Expertisepunt Mantelzorg	(Young) carer organization	https://www.mantelzorgers.be/nl	Tips, information, peer contact, fun activities
82	Hou me vast	Website with unclear origins	https://houmevast.nl/	Tips
83	Humanitas	Volunteer organization	https://www.humanitas.nl/	References, blogs/interviews, fun activities, vacations, buddy project, contact person
84	Ik heb dat	Patient or user organization	https://www.ikhebdat.nl/	References, blogs/interviews, information about diagnoses
85	Ikzorgvoor	(Young) carer organization	https://www.ikzorgvoor.com/	Tips, references, information, fun activities, gifts
86	Impuls	Social services	https://www.impulsaaenhunze.nl/	Fun activities, gifts
87	Incluzio Leiden	Social services	https://www.incluzioleiden.nl/	References, fun activities, contact person, information meetings and webinar for professionals
88	Indebuurt033	Social services	https://indebuurt033.nl/	References, group intervention, buddy project, gifts

89	Indigo	Specialized youth care	https://www.indigowest.nl/	Group intervention
90	Inevi	Network organization	https://inevi.nl/	Tips, references, information, peer contact
91	Inkendaal	Rehabilitation center or hospital	https://www.inkendaal.be/	Sibling day with a tour at the rehabilitation center
92	Inmovement	Charity	https://www.inmovement.nu/	Group intervention
93	Ipse de Bruggen	Disability care	https://www.ipsedebruggen.nl/	Family intervention that explicitly includes the siblings
94	Jeugdpreventie Amsterdam	Youth Social services	https://www.jeugdpreventieamsterdam.nl/	References, group intervention
95	JMZ Pro	(Young) carer organization	https://www.jmzpro.nl/	Tips, references, information, blogs/interviews, contact person, meetings and network for professionals
96	Joelle Riezebos	Private initiative	https://joelleriezebos.nl/	Blogs/interviews
97	Jong JGZ	Youth Social services	https://www.jongjgz.nl/	Information
98	Jonge Mantelzorg Vriendelijk School	(Young) carer organization	https://www.jongemantelzorgvriendelijkeschool.nl/	References, information, toolkit for schools
99	Kadodder	Disability care	https://www.kadodder.be/	Fun activities, group intervention, individual intervention (board game)
100	Karakter	Specialized youth care	https://www.karakter.com/	Group intervention
101	Karen Ten Wolde Coaching	Coach	https://karentenwolde.nl/	Information, individual intervention
102	Kearn	Social services	https://www.kearn.nl/	Fun activities, contact person, videos
103	Kennisplein Disability care	Knowledge organization	https://www.kennispleingehandicaptensector.nl/	Tips, references
104	Kentalis	Disability care	https://www.kentalis.nl/	Fun activities, group intervention

105	Kijk op welzijn	Social services	https://www.kijkopwelzijn.nl/	Tips, fun activities, family day, contact person
106	Kind & Ziekenhuis	Patient or user organization	https://kindenziekenhuis.nl/	Tips, references, information
107	Kind Zoekt Hulp	Private initiative	https://kindzoekthulp.nl/	References, information
108	Kind- en oudercoaching, Harderwijk en Ermelo	Coach	https://kindercoaching-harderwijk.nl/	References, information, group intervention, individual intervention
109	Kinderneurologie	Knowledge organization	www.kinderneurologie.eu	Tips, references
110	Kindertelefoon	Social services	www.kindertelefoon.nl	Contact person
111	Klik	Knowledge organization	https://www.klik.org/	Tips, references, information, blogs/interviews
112	Klimmendaal	Rehabilitation center or hospital	https://www.klimmendaal.nl/kinderen-jongeren	References
113	Koning Boudewijnstichting	Fund	https://kbs-frb.be/	Information, blogs/references, group interventions, individual interventions, recommendations for new projects
114	Koppelswoe	Social services	https://koppelswoe.nl/	Fun activities, group intervention, contact person, gifts
115	Kracht van beleving	Theatre group	https://www.krachtvanbeleving.nl/	References, videos, theatre play
116	Leekerweide	Disability care	https://www.leekerweide.nl/	References
117	Leieborg	Disability care	https://leieborg.be/	Peer contact, gifts
118	Lokaal Steunpunt Mantelzorg Gilze en Rijen	(Young) carer organization	https://www.jmzgilzerijen.nl/	Information, fun activities, contact person
119	Makkelijker Meedoen	Private initiative	https://makkelijkermeedoen.nl/	Blogs/interviews, book

120	Malkander	Social services	https://www.malkander-ede.nl/	References, information, fun activities, group intervention, buddy project, videos
121	Mama Vita	Patient or user organization	https://www.mamavita.nl/	Blogs/interviews
122	Mantelfoon	(Young) carer organization	https://mantelfoon.nl/	Tips, blogs/interviews, contact person
123	Manteling	(Young) carer organization	https://manteling.nu/	Information, fun activities, buddy project, gifts
124	Mantelzorg & Meer	(Young) carer organization	https://www.mantelzorgenmeer.nl/	Tips, references, information, fun activities, contact person
125	Mantelzorg Almelo	(Young) carer organization	https://www.mantelzorgalmelo.nl/	Fun activities, vacations, group intervention, buddy project, contact person
126	Mantelzorg Centraal	(Young) carer organization	https://www.mantelzorgcentraal.nl/	Tips, information, contact person, gifts
127	Mantelzorg Nijmegen	(Young) carer organization	https://mantelzorg-nijmegen.nl/	References, information, fun activities, contact person, gifts
128	Mantelzorg NL	(Young) carer organization	https://www.mantelzorg.nl/	Tips, references, information, blogs/interviews, “chill box”
129	Mantelzorgcentrum Nunspeet	(Young) carer organization	https://www.mantelzorg-nunspeet.nl/	Information, fun activities, videos
130	Mantelzorg- & Vrijwilligersplein (part of Sociom)	(Young) carer organization	https://mantelzorgenvrijwilligersplein.nl/	Information, blogs/interviews, gifts
131	Marieke Coacht	Coach	https://www.mariekecoacht-kindercoaching.nl/	Information, individual intervention
132	Markant	(Young) carer organization	https://www.markant.org/	References, information, group intervention, individual intervention, videos

133	MEE De Meent Groep	Client support organization	https://www.meedemeentgroep.nl/	Group intervention
134	MEE Dichtbij	Client support organization	https://www.mee-az.nl/	Peer contact, group intervention
135	MEE Gelderse Poort	Client support organization	https://www.meegeldersepoort.nl/	Group intervention
136	MEE Mantelzorg	(Young) carer organization	https://www.meemantelzorg.nl/	Information, blogs/interviews, fun activities, group intervention, gifts
137	MEE Rotterdam Rijnmond	Client support organization	https://www.meerotterdamrijnmond.nl/	Group intervention
138	MEEsamen	Client support organization	https://www.meesamen.nl/	Guest families
139	Mekanders	Disability care	https://www.mekanders.be/	Information
140	MENS	Social services	https://mensdichtbij.nl/	Contact person
141	Ministerie van Volksgezondheid, Welzijn en Sport	Ministry	https://www.rijksoverheid.nl/ministeries/ministerie-van-volksgezondheid-welzijn-en-sport	References, information
142	Minters	Social services	https://www.minters.nl/	Tips, information, contact person
143	Molenlanden	Youth Social services	https://www.molenlanden.nl/	Group intervention
144	MOmenz	Social services	https://momenz.nl/	Information
145	MPC Sint-Franciscus	Disability care	https://mpc-sintfranciscus.be/	Fun activities, vacations
146	MPC Terbank	Disability care	https://www.mpcterbank.be/	References, family days
147	MVT Arnhem	(Young) carer organization	https://www.mvtarnhem.nl/	Tips, references, information, fun activities, gifts

148	Nederlandse Vereniging voor Autisme	Patient or user organization	https://www.autisme.nl/	Tips, references, information, peer contact
149	Netwerk Jonge Mantelzorg Leidse Regio / Ikzorgvoor	(Young) carer organization	https://www.ikzorgvoor.com/	Tips, references, information, fun activities, gifts
150	Netwerk Ziezon	Care and education expertise network	https://ziezon.nl/	Tips, references, information
151	Nice2Bme	Coach	https://www.nice2bme.nl/	References, blogs/interviews, book, fun activities, individual interventions, e-learning for parents about siblings
152	Nederlands Jeugd Instituut	Knowledge organization	https://www.nji.nl/	Tips, references
153	Ons Stede Broec	Youth Social services	https://www.onsstedebroec.nl/	Information, fun activities
154	Ons Tweede Thuis	Disability care	https://www.onstweedethuis.nl/	Blogs/interviews
155	Op Koers	Rehabilitation center or hospital	https://opkoersonline.nl/	Group intervention
156	Op Weg Coaching	Coach	https://www.opwegcoaching.nl/	Individual intervention
157	Oro	Disability care	https://www.oro.nl/	Fun activities
158	Orthotalk40	Study project website	https://orthotalk40.wordpress.com/	References, information
159	Participate!	Knowledge organization	https://nl.participate-autisme.be/	Tips, information
160	Passie voor Jeugd en Gezin	Youth Social services	https://www.pjg.nu/	Family intervention that explicitly includes siblings
161	Pauline Kuiper	Private initiative	https://paulinekuiper.nl/	Tips, references, information, blogs/interviews, book
162	Philadelphia	Disability care	https://www.philadelphia.nl/	References

163	Prismanet	Disability care	https://www.prismanet.nl/	Blogs/interviews
164	Reinaerde	Disability care	https://www.reinaerde.nl/	Blogs/interviews, videos, family intervention that explicitly includes siblings
165	Resonans	Disability care	https://resonansvzw.be/	References, group intervention, respite care so the parent has time for the sibling
166	Revant	Rehabilitation center or hospital	https://www.revant.nl/	Opportunity for siblings to have a look at the therapy sessions
167	Rijndam	Rehabilitation center or hospital	https://www.rijndam.nl/	Information, references, group intervention
168	Ritmica	Disability care	https://www.ritmica.be/	Group intervention
169	Saam Welzijn	Social services	https://saamwelzijn.nl/	Peer contact, fun activities, individual intervention
170	Samana	Social services	https://samana.be/	Tips, references, information, blogs/interviews, fun activities, book, project focused on making schools ‘young carer friendly’
171	Same!	Disability care	https://www.wijzijnsame.nl/	Tips, references, information, blogs/interviews, peer contact
172	Schouders	Knowledge organization	https://schouders.nl/	Tips, references
173	Severinus	Disability care	https://www.severinus.nl/	Fun activities
174	's Heeren Loo	Disability care	https://www.sheerenloo.nl/	Blogs/interviews
175	Sien	Patient or user organization	https://www.sien.nl/	References, “sibling think tank”
176	Sint-Lievenspoort	Disability care	https://www.sintlievenspoort.be/	Vacations
177	Sociaal Werk de Kear	Social services	https://www.sociaalwerkdekear.nl/	Fun activities, contact person
178	Sociaal werk de Kop	Social services	https://www.sociaalwerkdekop.nl/	Information, fun activities, contact person

179	Sociaal werk de Schans	Social services	https://www.sociaalwerkdeschans.nl/	Tips, references, information, fun activities, contact person, videos
180	Sophi	Disability care	https://www.sophi.online/	Tips, references, information, blogs/interviews
181	STAN trefpunt verstandelijke handicap	Patient or user organization	https://www.trefpuntstan.be/	References
182	Start West-Vlaanderen	Disability care	https://www.startwestvlaanderen.be/	Information, fun activities, group intervention, individual intervention
183	Steunpunt Mantelzorg Soest	(Young) carer organization	https://zorgzaam.soest.nl/	References, information, blogs/interviews, fun activities, contact person, gifts
184	Steunpunt Mantelzorg Verlicht	(Young) carer organization	https://www.mantelzorgverlicht.nl/	References, information
185	Steunpunt Mantelzorg Zuid	(Young) carer organization	https://www.mantelzorgzuid.nl/nl	Information, blogs/interviews, fun activities, contact person, gifts
186	Steunpunt voor Mantelzorgers Parkstad	(Young) carer organization	https://www.mantelzorgparkstad.nl/	Information, blogs/interviews, fun activities, group intervention
187	Stichting ‘Ik ben een Brus’	Private initiative	https://ikbeneenbrus.nl/	References, information, fun activities
188	Stichting Bureau Sterrenstof	Charity	https://www.stichtingbureausterrenstof.nl/	Book
189	Stichting Dapper Kind	Charity	https://dapperdagboek.nl/	Book, worksheets, stickers
190	Stichting Down Syndroom	Patient or user organization	https://downsyndroom.nl/	Tips, references, information, blogs/interviews, books
191	Stichting Pulse	Social services	https://stichting-pulse.nl/	Tips, peer contact, fun activities, contact person, gifts

192	Stichting Sociaal Collectief	Social services	https://www.stichtingsociaalcollectief.nl/	References, information, fun activities
193	Stichting Tante Joy	Private initiative	https://www.tantejoy.nl/	Fun activities, vacations
194	Stichting Welzijn Hattem	Social services	https://www.swhattem.nl/	References, information, blogs/interviews, fun activities, contact person, gifts
195	Stichting Welzijnswerk	Social services	https://www.swwh.nl/	Fun activities, contact person
196	Stichting Wigwam	Charity that organizes vacations	https://www.stichtingwigwam.nl/	Blogs/interviews, vacations
197	Stichting ZO!	Social services	https://www.stzo.nl/	Fun activities, contact person
198	Stip	Social services	https://www.stipheerde.nl/	References, information, contact person, gifts
199	Strategische Alliantie Jonge Mantelzorg	(Young) carer organization	https://www.strategischealliantiejongemantelzorg.nl/	Tips, references, information, blogs/interviews, videos, conference, petition
200	Tandem	(Young) carer organization	https://www.tandemmantelzorg.nl/	Tips, references, information, blogs/interviews, contact person
201	Tanderuis	Disability care	https://www.tanderuis.be/	Fun activities, family day, group intervention, contact person
202	Theatergroep de Bint	Theatre group	https://theatergroepbint.nl/	Interactive education package
203	TiGO Gezinsondersteuning	Youth Social services	https://tigogezinsondersteuning.nl/	Tips, references, information, fun activities
204	Trimbos	Knowledge organization	https://www.trimbos.nl/	References, campaign
205	Universitair Medisch Centrum Groningen	Rehabilitation center or hospital	https://www.umcg.nl/	References, information
206	UP Foundation	Charity	https://upfoundation.nl/	References, conversation cards
207	Vaderklap	Private initiative	https://www.vaderklap.be/	Family day

208	Valtes	Private initiative	https://valtes.eu/	References, information
209	Vanzelfsprekend!?	(Young) carer organization	https://vanzelfsprekendjmz.nl/	Blogs/interviews, fun activities, group intervention, buddy project, contact person
210	Vera Duivenvoorden	Private initiative	https://veraduivenvoorden.nl/	Photo exposition
211	Vereniging Gehandicaptenzorg Nederland (VGN)	Branch association	https://www.vgn.nl/	References, blogs/interviews, videos, call for more research
212	Visio	Disability care	https://www.visio.org/home/	Peer contact
213	Vitis Welzijn	Social services	https://www.vitiswelzijn.nl/	Information, fun activities, group intervention, contact person
214	Vlaams Expertisepunt Mantelzorg	(Young) carer organization	https://www.mantelzorgers.be/	Tips, references, information, fun activities
215	VMCA Vrijwilligers en Mantelzorg Centrale Almere	(Young) carer organization	https://www.vmca.nl/	References, information, fun activities, group intervention, buddy project, contact person, videos
216	VTV	Charity	https://www.wijzijnvtv.nl/	Buddy project
217	VZW Victor	Disability care	https://www.vzwvictor.be/	Group intervention
218	Wat Niemand Weet	Private initiative	https://wat-niemand-weet.nl/	Blogs/interviews, book
219	Watwat	Information platform	https://www.watwat.be/	Tips, references, blogs/interviews
220	Week van de Jonge Mantelzorger	(Young) carer organization	https://www.weekvandejongemantelzorger.nl/	Tips, references, information, blogs/interview, campaign videos
221	Wegwijs	Disability care	https://www.wegwijslimburg.be/	Tips, references, information, group intervention, individual intervention, outreach for professionals, attention from family caregiver
222	Welzijn Barneveld	Social services	https://www.welzijnbarneveld.nl/	References, information, fun activities, contact person

223	Welzijn Capelle	Social services	https://www.welzijncapelle.nl/	Information, contact person
224	Welzijn in Noordenveld	Social services	https://www.welzijninnoordenveld.nl/	Tips, references, fun activities, buddy project, contact person
225	Welzijn Lelystad	Social services	https://www.welzijnlelystad.nl/	Tips, references, fun activities, vacations, group intervention, individual intervention, buddy project, contact person, gifts
226	Welzijn Lochem	Social services	https://welzijnlochem.nl/	Information, fun activities, individual intervention, buddy project, contact person, gifts
227	Welzijn MensenWerk	Social services	https://www.welzijnmw.nl/	Tips, references, information, fun activities, contact person
228	Welzijn Noordwijk	Social services	https://www.welzijnnoordwijk.nl/	References, information, contact person
229	Welzijn Rijswijk	Social services	https://welzijn-rijswijk.nl/	Tips, information, fun activities, contact person, gifts
230	Welzijn Teylingen	Social services	https://www.welzijnteylingen.nl/	References, information, contact person, gifts
231	Welzijn West Betuwe	Social services	https://welzijnwestbetuwe.nl/	Tips, information, fun activities, contact person, gifts
232	Welzijn Wolden	Social services	https://welzijndewolden.nl/	Fun activities, contact person
233	Welzijn Wonen Plus	Social services	www.welzijnwonenplus.nl	Fun activities
234	Welzijnshuis Borsele	Social services	https://www.welzijnshuisborsele.nl/	Blogs/interviews, fun activities, gifts
235	Welzijnskwartier	Social services	https://welzijnskwartier.nl/	References, fun activities, gifts
236	Welzijnswerk Midden Drenthe	Social services	https://www.welzijnswerkmd.nl/	Gifts
237	Wijkracht Hengelo	Social services	https://www.wijkrachthengelo.nl/	Buddy project, contact person
238	Wijkteams Enschede	Social services	https://www.wijkteamsenschede.nl/	Group intervention
239	Wijzijn	Social services	https://www.wijzijn.nl/	Fun activities

240	Woej	Charity	https://www.woej.nl/	Fun activities
241	Wolkenkaarten	Patient or user organization	https://wolkenkaarten.nl/	Tips, information, videos, conversation cards
242	WUH Winterwijks	Volunteer organization	https://wuhwinterswijk.nl/	Vacations
243	Xonar	Youth Social services	https://www.xonar.nl/	Blogs/interviews, family intervention that explicitly includes siblings
244	Xtra	Youth Social services	https://xtra.nl/	Contact person
245	Yfke	Social services	https://wijzijnyfke.nl/	Tips, information, blogs/interviews, peer contact, fun activities, vacations, buddy project, contact person, gifts
246	YMCA Nederland	Patient or user organization	https://ycamps.nl/	Vacations
247	Ypse	Social services	https://ypse.nl/	Information, group intervention, individual intervention
248	Yulius	Specialized youth care	https://www.yulius.nl/	Group intervention
249	Zitdazo	Private initiative	https://www.zitdazo.be/	Individual intervention
250	Zo Jong	(Young) carer organization	https://www.zojong.be/	Tips, references, information, blogs/interviews
251	ZonMw	Knowledge organization	https://www.zonmw.nl/	References, blogs/interviews
252	Zorg Welzijn	Knowledge organization	https://www.zorgwelzijn.nl/	Blogs/interviews
253	Zorgdat	Social services	https://zorgdat.nl/	Fun activities, contact person, gifts
254	Zuyderland	Rehabilitation center or hospital	https://www.zuyderland.nl/	Group intervention
255	Zwolle Doet!	Volunteer organization	https://zwolledoet.nl/	Tips, information, blogs/interviews, fun activities, group intervention, buddy project, contact person

Table S4.2

Included Books (in alphabetical order)

#	Name (*translation*)	Type	Author(s)	Publisher	Year of publication
1	Alle vragen over Downsyndroom *(All questions about Down Syndrome)*	Informative book for siblings	Lamberts, R. & De Graaf, G.	Stichting Downsyndroom	*Unknown*
2	Altijd weer wat! Belevenissen van een brusje (*Always something going on! Experiences of a sibling*)	Youth book (fiction)	Puts, F.	Pica	2008
3	Bijzondere broers en zussen (*Special brothers and sisters*)	Informative book for professionals and parents	Hames, A. & MacCaffrey, M.	Niño	2006
4	BOZ-boek *(Brother-or-Sister-book)*	Activity book	*Unknown*	Stichting Dapper Kind	2023
5	Broers en zussen van speciale en gewone kinderen *(Brothers and sisters of special and ordinary children)*	Informative book for professionals and parents	Boer, F.	Lannoo Campus	2021
6	Broers en zussen: hoe is het voor jou? *(Brothers and sisters: what is it like to you?)*	Book with personal stories	Sasabone, E.	Bureau Sterrenstof	2018
7	Broers- en zussenboek (*Brothers and sisters book*)	Book with personal stories and tips	Van Dijken, A.	Lannoo Campus	2013

8	Broers- en zussenboek in en om het ziekenhuis (*Brothers and sisters book in and around the hospital*)	Activity book	Van Dijken, A.	Lannoo Campus	2017
9	Brus en zus: samen bijzonder (*Sibling and sister: special together*)	Story/picture book	Kaars, S.	Uitgeverij Licht	2020
10	BRUS, (on)breekbare liefde (*SIBLING, (un)breakable love*)	Book with personal stories, pictures and letters from siblings	Kuiper, P	Scholten Uitgeverij	2019
11	Brussen in beeld (*Siblings in the picture*)	Informative book for parents	Hoedemaekers, N.	*Not applicable*	2024
12	De liefste of de dapperste of allebei (*The dearest or the bravest or both*)	Youth book (fiction)	Franck, E.	Davidsfonds	2010
13	De Pizzabende (*The Pizza gang*)	Youth book (fiction)	Jacobs, A	Leopold	2022
14	De wereld van Luuk (*Luke's world*)	Story/picture book	Delfos, M.F.	SWP	2014
15	Dex+Zo, de snoezelbrigade - stop pesten! (*Dex&co, the snoozle squad – stop bullying!)*	Story/picture book	Budding-Corbée, J.	Stichting de Dex Foundation	2011
16	Dus ik ben een brus?! (*So, I am a sibling?!)*	Youth book (fiction)	Bruessing, L.N.	Bruessing	2018

17	Een broertje anders dan anders (*A brother different than others*)	Story/picture book	Delval, M.	Start Brugge	1993
18	Een huis om in te verdwalen *(A house to get lost in)*	Youth book (fiction)	Vandermeeren, H.	Davidsfonds	2002
19	Fien kan niet goed zien (*Fien cannot see well*)	Story/picture book	Joosen, W.	Boekscout	2016
20	Getraumatiseerde kinderen met een verstandelijke beperking: Helpen bij herstel (*Traumatized children with an intellectual disability: Help with recovery*)	Informative book for professionals	Scharloo, A., Van Kregten, C. & Soro, G.	Bohn Stafleu van Loghum	2021
21	Gewone jongeren, bijzondere jeugd (*Normal youngsters, special youth*)	Book with personal stories and pictures	*Unknown*	Stichting de Kap	2022
22	Gewoon daarom (*Just because*)	Story/picture book	Elliott, R.	C. de Vries-Brouwers	2012
23	Groene appels en zussenknuffels (*Green apples and sister cuddles*)	Book with personal stories	Poppe, L., Steel, R. & Vandevelde, S.	Academia Press	2009
24	Help, het brusje is normaal (*Help, the sibling is normal*)	Informative book for parents	Vossen, T.	Graviant Bv	2012
25	Help, mijn zus is gek (*Help, my sister is crazy*)	Youth book (fiction)	Van Kempen, F.	Clavis Uitgeverij	2010

26	Het is oké... Een boek voor brussen (*It's okay… A book for siblings*)	Story/picture book	Bruyenberg, L.	Brave New Books	2018
27	Hildeke (*Little Hilde*)	Adult book (personal experiences of a sibling)	Joris, L.	Atlas Contact	2022
28	Iedereen is anders geboren (*Everyone is born different*)	Story/picture book	De Jong, N.	B publishing	2014
29	Ik ben er ook nog! (*I am also there!*)	Book with personal stories and tips	Quadackers, F.	Brave New Books	2016
30	Jij kunt toch niets! Ode aan mijn autistische broer (*You can't do anything! Ode to my autistic brother*)	Adult book (personal experiences of a sibling)	Poot, E.	Uitgeverij Aspekt	2021
31	Jonge mantelzorgers op school (*Young carers at school*)	Informative book for professionals	Vanderlinden, J. & Van Walle, K.	Politeia	2019
32	Josefientje Mongolientje (*Josephine Mongoline*)	Youth book (fiction)	Verroen, D.	Unieboek Het Spectrum	2003
33	Len en zijn broer (*Len and his brother*)	Story/picture book	Wilms, M.	Leiderschapsdomeinen	2019
34	Liever dan lief: Een sprookje in het land van Down (*Sweeter than sweet: A fairytale in the land of Down*)	Story/picture book	Jonker, B.	De Vier Windstreken	2020

35	Mag ik ook ff?<br>(*Can I have a moment too?*)	Activity book	Kapitein, M. & Van der Horst, R.	De Hersenstichting	2009
36	Mick is anders, omdat hij autisme heeft<br>(*Mick is different, because he has autism*)	Story/picture book	De Bruin, C. & De Bruin, A.	Graviant Bv	2009
37	Mijn broer heeft autisme<br>(*My brother has autism*)	Informative book for siblings with tips for parents	Kaptein, E.	248media Uitgeverij	2020
38	Mijn broer is een marsmannetje<br>(*My brother is a martian*)	Youth book (fiction)	Minne, B.	Clavis B.V.B.A.	1993
39	Mijn broer is een orkaan<br>(*My brother is a hurricane*)	Youth book (fiction)	Janssen, K.	Davidsfonds	1994
40	Mijn broertje is bijzonder<br>(*My brother is special*)	Story/picture book	Vandaele, A. & Bongini, B.	Clavis	2022
41	Mijn gehandicapte broer & ik<br>(*My disabled brother & I*)	Comic book	Derkx, C.	Camiel Derkx	2018
42	Mijn gehandicapte broer & ik, deel twee<br>(*My disabled brother & I, part 2*)	Comic book	Derkx, C.	Camiel Derkx	2021
43	Mijn knettergekke zus<br>(*My crazy sister*)	Youth book (fiction)	Selmer, A.	Lannoo	2000

44	Mijn zus heeft autisme<br>(*My sister has autism*)	Informative book for siblings with tips for parents	Kaptein, E.	248Media Uitgeverij	2020
45	Mijn zus is een flussemus<br>(*My sister is a 'flussemus'*)	Story/picture book	Van Duyn, S.	Van Goor	2002
46	Mijn zus woont in het donker<br>(*My sister lives in the dark*)	Story/picture book	Geyskens, E.	Altiora Averbode	2014
47	Mijn zusje heeft Downsyndroom<br>(*My sister has Down Syndrome*)	Story/picture book	Doppen, J.	Uitgeverij Pica	2013
48	Mijn zusje is anders<br>(*My sister is different*)	Story/picture book	Van Andel, L.	De Ruiter	1997
49	Mikko, mijn stoere broer<br>(*Mikko, my tough brother*)	Story/picture book	Vink, H.	Uitgeverij Johannes Multimedia	2003
50	Roosje en Schildpad<br>(*Rosie and Turtle*)	Story/picture book	Wild, M. & Brooks, R.	Middernacht Pers	1999
51	Salto<br>(*Somersault*)	Youth book (fiction)	Thórarinsdóttir, A.	Volt	2024
52	Sander en Anders<br>(*Sander and Anders*)	Story/picture book	De Coensel, L.	De Kangoeroe Vzw	2012
53	Stijn is anders<br>(*Stijn is different*)	Story/picture book	Vanvuchelen, M.	Lannoo Uitgeverij	2002

54	Superbrus, een megaklus (*Super sibling, a huge task*)	Informative book for professionals	Snoeckx, A.	Garant Uitgevers	2012
55	Tinus, gewoon mijn zusje (*Tinus, just my sister*)	Adult book (personal experiences of a sibling)	Wolting-Hulzinga, N.	*Not applicable*	2019
56	Trampoline naar de hemel (*Trampoline to heaven*)	Youth book (fiction)	Vermeulen, E.	Averbode	2002
57	Tussen trots en ergernis: als je broer of zus autisme heeft (*Between pride and annoyance: when your brother or sister has autism*)	Book with personal stories	Van der Linden, E.	Uitgeverij Epo	2015
58	Van hier tot aan de maan en terug (*To the moon and back*)	Adult book (personal experiences of a parent)	Boon, E.	Houtekiet	2014
59	Vragen voor en door brussen (*Questions for and from siblings*)	Activity book	Steel, R., Moyson, T., Vandevelde, S. & Poppe, L.	Hogeschool Gent	2009
60	Wachten op een snor (*Waiting for a moustache*)	Youth book (fiction)	De Doncker, W.	Davidsfonds	1993
61	Wat is het: het Syndroom van Down (*What is it: Down Sydrome*)	Informative book for siblings	Royston, A.	Ars Scribendi B.V. H.O.D.N. Schoolsuppor	2008
62	Wat niemand weet (*What no one knows*)	Adult book (personal experiences of parents)	Van der Velde, E., Warmerdam, S. & Werz, V.	Lotje&co	2019

63	Weet jij wat autisme is?<br>(*Do you know what autism is?*)	Activity book	Sabin, E.	Uitgeverij Pica	2018
64	Weet jij wat het is om anders te zijn?<br>(*Do you know what it is like to be different?*)	Activity book	Sabin, E.	Uitgeverij Pica	2007
65	Zus van broer<br>(*Sister of brother*)	Youth book (fiction)	Van Weelden, M. & Tiddens, P.	Eigen Werk Theaterteam	2023

Table S4.3

Included Media Sources (in alphabetical order)

#	Name (*translation*)	Type	Broadcaster/ organization	Year of publication	URL
1	Bikkels (*Tough cookies*)	TV series	VPRO	2013- 2015	https://www.vpro.nl/jeugd/programmas/bikkels.html
2	Bikkels educatieclips (*Tough cookies educational clips*)	Educational clips	VPRO	*Unknown*	http://www.bikkelseducatie.nl/
3	Brussen (*Siblings*)	TV talkshow	MAX	2016	https://www.maxvandaag.nl/sessies/themas/familie-relatie/brussen/
4	Congres Jonge Mantelzorg: opening - ervaringsverhaal Gino (*Young Carers Conference: opening – personal story Gino*)	YouTube video	Strategische Alliantie Jonge Mantelzorg	2021	https://www.youtube.com/watch?v=X70TGtvMtDk&rco=1&ab_channel=StrategischeAlliantieJongeMantelzorg
5	De broer van Josephine is ernstig gehandicapt (*The brother of Josephine has a severe disability*)	Youth news episode	NOS	2019	https://www.youtube.com/watch?v=x6ciMfMT9hE&ab_channel=NOSJeugdjournaal
6	Die heeft een gek broertje zeg! (*He's got a weird brother!*)	YouTube video	GGZ Eindhoven	2014	https://www.youtube.com/watch?v=4MKo96kJ-RY&ab_channel=tijdcode
7	Elin (*Elin*)	Documentary	NPO	2022	https://www.npodoc.nl/documentaires/2022/08/Elin.html
8	Hoofdzaken (*Main business*)	TV series episode	NPO	2022	https://npo.nl/start/serie/hoofdzaken/seizoen-1/rayan-ivy-olivia-zeccas-milan/afspelen

9	Ik ben een jonge mantelzorger (*I am a young carer*)	Documentary	GLD	2022	https://www.gld.nl/tv/aflevering/gld-doc-ik-ben-jonge-mantelzorger/170649
10	In gesprek met brussen (*In conversation with siblings*)	YouTube video	University of Humanistic Studies	2024	https://www.youtube.com/watch?v=EUkwq31EzoA
11	Jij ziet, jij ziet wat ik niet zie (*You see, you see what I don't see*)	TV series episode	NPO	2024	https://npo.nl/start/serie/jij-ziet-jij-ziet-wat-ik-niet-zie/seizoen-1/jij-ziet-jij-ziet-wat-ik-niet-zie/afspelen
12	Jong geleerd (*What is learned young*)	Documentary	Ethics filmservice	2022	https://ethicsfilmservice.com/film/e22fcf
13	Jonge mantelzorgers in gesprek met minister Hugo de Jonge (*Young carers in conversation with minister Hugo de Jonge*)	YouTube video	Ministry of Health, Welfare and Sport	2020	https://www.youtube.com/watch?v=KsZLMaj-lcA&ab_channel=MinisterievanVolksgezondheid%2CWelzijnenSport
14	Langs de Lijn En Omstreken aflevering: "Iris Hoogendoorn maakt serie over broer met beperking" (*Along the Line and Surroundings: "Iris Hoogendoorn makes a series about a brother with a disability*)	Radio talkshow	NPO	2021	https://www.nporadio1.nl/fragmenten/langs-de-lijn-en-omstreken/f2d4c6f5-0e8d-4959-8325-552ed511a3cf/2021-01-21-iris-hoogendoorn-maakt-serie-over-broer-met-beperking
15	Mijn broeders hoeder (*My brother's keeper*)	Documentary	NPO	2018	https://www.npodoc.nl/documentaires/2018/10/mijn-broeders-hoeder.html
16	Mijn Brus Verhaal (*My Sibling Story*)	Instagram page	Private source	2021-now	https://www.instagram.com/mijn_brus_verhaal/
17	Mijn kleine grote broer (*My little big brother*)	TV series	NPO	2021	https://npo.nl/start/serie/mijn-kleine-grote-broer

18	Onbekend maak onbemind - Ik zou mijn gehandicapte broer nooit anders willen dan hoe hij nu is (*Unkown makes unloved – I wouldn't want my disabled brother to be any other way than he is now*)	Podcast episode	Frion zorg	2022	https://open.spotify.com/episode/6qIiF5wk0QBm8OSo9vVeVt
19	Op zoek naar mijn zusje (*Searching for my sister*)	YouTube video	Private source	2016	https://www.youtube.com/watch?v=Dn1GMOKfA6w
20	Schatje patatje (*Sweetie-pie*)	Vimeo video	BRUS	2018	https://vimeo.com/667179667
21	Summerparty voor jonge mantelzorgers in Ommen (*Summerparty for young carers in Ommen*)	YouTube video	Vechtdal TV	2023	https://www.youtube.com/watch?v=aOpTN-Qi3LE&ab_channel=VechtdalTV
22	Van 0 tot 23 (*From 0 to 23*)	YouTube video	RTV Rijnmond	2011	https://www.youtube.com/watch?v=BegCJBLIUaU&ab_channel=RTVRijnmondExtra
23	Villa VdB (*Villa VdB*)	Podcast episode	NPO	2023	https://www.nporadio1.nl/fragmenten/villa-vdb/655e2355-7c17-4047-95b8-f66af62741c1/2023-06-05-een-op-de-vier-jongeren-is-mantelzorger-moeten-zichzelf-niet-te-veel-wegcijferen
24	Waar de helden zijn (*Where the heroes are*)	Documentary	EigenWerk Theaterteam	2022	https://vimeo.com/599587321
25	Wat zou jij doen? Berber (*What would you do? Berber*)	TV series episode	ZAPP	2023	https://www.zapp.nl/programmas/1892-wat-zou-jij-doen/gemist/VPWON_1350040

26	Wat zou jij doen? Julian en Constantijn (*What would you do? Julian and Constantijn*)	TV series episode	ZAPP	2019	https://www.zapp.nl/programmas/1892-wat-zou-jij-doen/gemist/VPWON_1302885
27	Zie je mij? (*Do you see me?)*	Podcast	Nivoz	2021	https://nivoz.nl/nl/podcast-zie-je-mij
28	Zin in morgen (*Looking forward to tomorrow*)	TV series episode	NPO	2024	https://npo.nl/start/serie/zin-in-morgen/seizoen-3/zin-in-morgen_47/afspelen
29	Zomervacht (*Summer Brother*)	Movie	Family Affair Films	2023	https://septemberfilm.nl/films/1090-Zomervacht
30	'Zorgen voor mijn broer? Dat kon ik helemaal niet' (*Taking care of my brother? I couldn't do that at all*)	Radio talkshow	NPO	2021	https://www.human.nl/de-publieke-tribune/artikelen/zorgen-voor-mijn-broer-dat-kon-ik-helemaal-niet

Table S4.4

Included News Articles (in alphabetical order)

#	Name (*translation*)	Newspaper/ magazine	Type	Year of publication	URL
1	Aandacht voor gezinsleden ernstig beperkt kind noodzakelijk (*Attention for family members of severely disabled child needed*)	Blik op Hulp	Online platform	2016	https://blikophulp.nl/gezinsleden-ernstige-verstandelijke-beperking-meervoudige-beperking-jorien-luijxk/
2	Berber Tieman, jonge mantelzorger van elf jaar (*Berber Tieman, young carer of eleven years old*)	Gezond & Fit van Groot Heerenveen	Regional newspaper	2022	https://www.jmzpro.nl/wp-content/uploads/2023/01/GH2210-Caleidoscoop.pdf
3	Brusjes moeten veel incasseren en opofferen (*Siblings have to put up with and sacrifice a lot*)	Autisme magazine	Professional journal	2021	https://www.autisme.nl/wp-content/uploads/2025/02/AM0221_Brusjes.pdf
4	Brusje-zijn (*Being a sibling*)	Rino Groep	Blog of education center	2018	https://www.rinogroep.nl/blog/268/brusje-zijn.html
5	Den Bosch gaat praten met schoolleiding over jonge mantelzorgers (*Den Bosch will talk with school directors about young carers*)	DTV Nieuws	Regional newspaper	2024	https://dtvnieuws.nl/nieuws/artikel/den-bosch-gaat-praten-met-schoolleiding-over-jonge-mantelzorgers
6	Een gehandicapte broer: Ben ik zijn zus of zijn verzorger? (*A disabled brother: Am I his sister or his carer?*)	Het Financieel Dagblad	National newspaper	2022	https://fd.nl/samenleving/1451783/een-gehandicapte-broer-ben-ik-zijn-zus-of-verzorger-j2j2caEImUzA

7	Fien heeft een kleine grote broer (*Fien has a little big brother*)	Kidsweek	National newspaper for children	2021	https://www.kidsweek.nl/nieuws/fien-heeft-een-kleine-grote-broer~bcfbcb7
8	Ik zat in een overlevingsstand, al mijn werkelijke gevoelens stopte ik weg (*I am in survival mode, I pushed away all my real feelings*)	EO	Public broadcaster	2021	https://www.eo.nl/artikel/ik-zat-in-een-overlevingsstand-al-mijn-werkelijke-gevoelens-stopte-ik-weg
9	Isabella (17): “Ik had pas door dat ik jonge mantelzorger was, toen ik een pakketje ontving” (*Isabella (17): “I only knew I was a young carer when I received a parcel”*)	Omroep Lingewaard	Regional broadcaster	2023	https://omroeplingewaard.nl/isabella-17-ik-had-pas-door-dat-ik-jonge-mantelzorger-was-toen-ik-een-pakketje-ontving/
10	Jolijn (11) is mantelzorger voor haar zusje: 'Ik mag niet hard lachen' (*Jolijn (11) is young carer of her sister: ‘I cannot laugh loudly’*)	Omroep Gelderland	Regional broadcaster	2022	https://www.gld.nl/nieuws/7700677/jolijn-11-is-mantelzorger-voor-haar-zusje-ik-mag-niet-hard-lachen
11	Jonge mantelzorgers bakken taart en koekjes bij Bakker De Jager (*Young carers bake cakes and cookies at Baker De Jager*)	Het Kontakt Vijfheerenlanden	Regional newspaper	2024	https://www.hetkontakt.nl/vijfheerenlanden/nieuws/390146/jonge-mantelzorgers-bakken-taart-en-koekjes-bij-bakker-de-jager
12	Jonge mantelzorgers leven zich uit in ‘modderpoel’ (*Young carers let off steam in the ‘mud puddle’*)	De Mooi Son en Breugel Krant	Regional newspaper	2024	https://www.mooisonenbreugel.nl/algemeen/algemeen/37094/jonge-mantelzorgers-leven-zich-uit-in-modderpoel
13	Leuke activiteiten voor jonge mantelzorgers (*Fun activities for young carers*)	Omroep Hoeksche Waard	Regional broadcaster	2024	https://www.omroephw.nl/2024/06/leuke-activiteiten-voor-jonge-mantelzorgers/

14	Lisa’s zus heeft een verstandelijke beperking (*Lisa’s sister has an intellectual disability*)	Vriendin	Lifestyle magazine	2023	https://www.vriendin.nl/persoonlijke-verhalen/lisas-zus-verstandelijke-beperking/
15	Mantelzorger Naomi (11) uit Hoogerheide zorgt voor haar zusje: ‘Maar ik heb ook tijd voor hobby's’ (*Young carer Naomi (11) from Hoogerheide cares for her little sister: ‘But I also have time for hobbies’*)	BN De Stem	National newspaper	2023	https://www.bndestem.nl/nieuws/mantelzorger-naomi-11-uit-hoogerheide-zorgt-voor-haar-zusje-maar-ik-heb-ook-tijd-voor-hobby-s~a4c17a84/
16	Marilynn (9) zorgt voor haar grote zus: 'Als ik haar help, maakt dat haar blij' (*Marilynn (9) takes care of her big sister: ‘When I help her, that makes her happy’*)	RTV Noord	Regional broadcaster	2024	https://www.rtvnoord.nl/zorg/1171497/marilynn-9-zorgt-voor-haar-grote-zus-als-ik-haar-help-maakt-dat-haar-blij
17	Ons Stede Broec organiseert Brusjesmiddag op 26 april (*Ons Stede Broec organizes a Sibling afternoon on April 26th*)	Ons West-Friesland	Regional broadcaster	2023	https://onswestfriesland.nl/2023/nieuws_stedebroec/brusjesmiddag-26-april/
18	Opgroeien met een zieke of gehandicapte broer of zus: "Ik zal altijd voor je zorgen" (*Growing up with a sick or disabled brother or sister: “I will always take care of you”*)	Libelle	Lifestyle magazine	2019	https://www.libelle.nl/actueel/opgroeien-met-een-zieke-of-gehandicapte-broer-of-zus-ik-zal-altijd-voor-je-zorgen~bd947f99/
19	Week van de jonge mantelzorger: Een interview met Eva Bak (14 jaar) (*Young Carer’s Week: An interview with Eva Bak (14 years*)	Gemeente Opmeer	Municipality	2024	https://www.opmeer.nl/jonge-mantelzorger-eva
20	Week van de Jonge Mantelzorger: meer aandacht voor impact mantelzorg (*Young Carer’s Week: More attention for impact of caring*)	Sleutelstad	Regional broadcaster	2023	https://sleutelstad.nl/2023/05/19/week-van-de-jonge-mantelzorger-meer-aandacht-voor-impact-mantelzorg/

Table S4.5

Included Research Texts and Reports (in alphabetical order)

#	Name (*translation*)	Type	Author(s)	Organization/ University	Year of publication	URL
1	Bezorgd naar school. Kwaliteit van leven van scholieren met een langdurig ziek gezinslid *(Going to school worried. Quality of life of pupils with a chronically ill family member)*	Report	De Roos, S., Van Tienen, I. & De Boer, A.	Netherlands Institute for Social Research	2020	https://www.jmzpro.nl/wp-content/uploads/2020/07/feb-2020-SCP-Bezorgd-naar-school.pdf
2	Brus - en dan…? Een onderzoek naar de betekenis van het hebben van een broer of zus met een verstandelijke beperking (*Sibling – now what…? A study into the meaning of having a brother or sister with an intellectual disability*)	Thesis	Vermeer, F.	Tilburg University	2011	https://arno.uvt.nl/show.cgi?fid=115254
3	Family matters: The experiences and opinions of family members of persons with (severe) or profound intellectual disabilities.	Dissertation summary	Luijkx, J.	Groningen University	2016	https://pure.rug.nl/ws/portalfiles/portal/36109193/Samenvatting.pdf
4	Gevolgen van coronamaatregelen voor naasten van mensen met een verstandelijke beperking: Onderzoek naar kwaliteit van leven tijdens de tweede golf. (*Consequences of corona regulations for next of kin of*	Report	Boeije, H., Van Schelven, F. & Verkaik, R.	Nivel	2021	https://www.nivel.nl/nl/publicatie/gevolgen-van-coronamaatregelen-voor-naasten-van-mensen-met-een-verstandelijke-beperking

	persons with an intellectual disability: Study into the quality of life during the second wave)					
5	Interactieverschillen tussen de brusrelatie en de broerzusrelatie binnen één gezin (*Interaction differences between sibling relationships with the sibling with or without a disability within one family*)	Thesis	Roosen, M.	Gent University	2010	https://libstore.ugent.be/fulltxt/RUG01/001/460/318/RUG01-001460318_2011_0001_AC.pdf
6	Kan een serious game het welzijn van brussen van 6-9 jaar met een broer of zus met een visuele en/of verstandelijke beperking vergroten? (*Can a serious game improve the well-being of siblings age 6-9 years with a brother or sister with a visual impairment or intellectual disability?*)	Poster	Veerman, L., Van Dijken, A., Willemen, A., Derks, S. & Sterkenburg, P.	Vrije Universiteit Amsterdam	2022	https://viafutura-production-uploads.s3.eu-west-1.amazonaws.com/public/gallery/wrUjZMaCZjHAXn41PHVe/629711932d4f2/documents/CzVT36S3IeNQRUtr.pdf
7	Kwalitatief onderzoek naar de ondersteuningsbehoefte van brussen binnen Stichting Abrona (*Qualitative research into the support needs of siblings within Stichting Abrona*)	Thesis	Klomp, S.	NTI Leiden	2017	https://brussensite.wordpress.com/wp-content/uploads/2017/08/kwalitatief-onderzoek-brussen-stichting-abrona-20162017.pdf

8	Kwaliteit van leven van naasten van mensen met een beperking: Publiekssamenvatting. (*Quality of life of next of kin of persons with disabilities: Public summary*)	Summary of a report	Boeije, H. & Van Schelven, F.	Nivel	2020	https://www.nivel.nl/nl/publicatie/kwaliteit-van-leven-van-naasten-van-mensen-met-een-beperking-publiekssamenvatting
9	Literatuurverkenning kwaliteit van leven van naasten: Studies van 2018-2022 samengebracht als opstap voor vervolgonderzoek. (*Literature review quality of life of next of kin: Studies from 2018-2022 brought together as a step towards future research*)	Report	Zonneveld, E., Scherpenzeel, A. & Boeije, H.	Nivel	2022	https://www.nivel.nl/nl/publicatie/literatuurverkenning-kwaliteit-van-leven-van-naasten-studies-van-2018-2022-samengebracht
10	Mijn broer is autistisch, maar wie ben ik? Onderzoek naar de ervaringen van een brusje (*My brother is autistic, but who am I? Research into the experiences of a sibling*)	Thesis	De Jong, A.	Fontys OSO	2012	https://hbo-kennisbank.nl/details/sharekit_fontys:oai:surfsharekit.nl:4bff20a8-20fb-470f-b2ad-364bfec33de8?has-link=yes&c=0&q=broer&p=2
11	Onderzoek naar het gebruik van discoursen rondom mensen met een beperking gezien vanuit broers en zussen en de rol die sport hierin speelt (*Research into the use of discourse around people with a disability seen from their brothers' and sisters' perspectives*	Thesis	Van der Westen, A.	Utrecht University	2016	https://studenttheses.uu.nl/bitstream/handle/20.500.12932/25269/Masterscriptie%20Aniek%20van%20der%20Westen1.pdf?sequence=1

	and the role that sport plays in this)					
12	Opgroeien met zorg: Quick scan naar de aard en omvang van zorg, belasting en ondersteuningsmogelijkheden voor jonge mantelzorgers (*Growing up in a situation of care: Quick scan into the nature and scope of care, burden and support possibilities for young carers*)	Report	De Veer, A.J.E. & Francke, A.L.	Nivel	2008	https://www.nivel.nl/sites/default/files/bestanden/Rapport-Quick-scan-aard-en-omvang-zorg-jonge-mantelzorgers.pdf
13	Oudere broers en zussen van mensen met een verstandelijke beperking. Een groepsportret (*Older brothers and sisters of persons with an intellectual disability. A group portret*)	Thesis	Van Dijk, J. & Jongerius, P.	*Unknown*	*Unknown*	https://core.ac.uk/download/pdf/39700662.pdf
14	Pedagogiekstudent ontwerpt familiespel voor families met verstandelijk beperkt kind (*Pedagogy student designs a family game for families of children with intellectual disabilities*)	Study project	Buijnsters, J.	Fontys University of Applied Sciences	2016	https://www.fontys.nl/nieuws/pedagogiekstudent-ontwerpt-familiespel-voor-families-met-verstandelijk-beperkt-kind/
15	Quickscan jonge mantelzorgers, 2007 (*Quickscan young carers, 2007)*	Report	De Veer, A.J.E. & Francke, A.L.	Nivel	2008	https://www.nivel.nl/nl/project/quickscan-jonge-mantelzorgers-2007

16	QuickScan naar de ondersteuningsbehoefte van zorgintensieve gezinnen. Visiedocument deel 2: Brussen (*Quickscan into the support needs of families with intensive care. Vision document, part 2: Siblings*)	Report	Okma, K., Van Dijken, A.,Vergeer, M. & Naafs, L.	Netherlands Youth Institute	2015	https://static1.squarespace.com/static/617d0df06ddae414a521778e/t/61d9a72a0c377c7dc3e09260/1641654060447/2015+NJi+-+QuickScan+ondersteuningsbehoefte+zorgintensieve+gezinnen+deel+2+Brussen.pdf
17	Samen sterk! Een onderzoek naar een eenduidige aanpak voor het betrekken van brusjes bij de behandeling binnen IBC-kind. (*Stronger together! A study into the current approach of involving siblings in the treatment within IBC-child*)	Thesis	Van der Sluijs, R. & Scholten, M.	University of Applied Sciences Arnhem and Nijmegen	2016	https://hbo-kennisbank.nl/details/sharekit_han:oai:surfsharekit.nl:32cde87f-708b-4318-b562-bd5a2ea0e6d0
18	Volwaardig leven *(Fulfilling life)*	Report with portraits	*Unknown*	National government	2020	https://open.overheid.nl/documenten/ronl-3160fcc7-cf49-4dcf-8318-1d6f1f112d75/pdf
19	Wat werkt bij de ondersteuning van jonge mantelzorgers (*What works in supporting young carers*)	Report	Booijink, M., & De Haan, J.	Movisie	2020	https://www.movisie.nl/sites/movisie.nl/files/2020-06/Wat-werkt-bij-dossier-Wat-werkt-bij-de-ondersteuning-van-jonge-mantelzorgers.pdf
